# MEDYAN: Mechanochemical Simulations of Contraction and Polarity Alignment in Actomyosin Networks

**DOI:** 10.1371/journal.pcbi.1004877

**Published:** 2016-04-27

**Authors:** Konstantin Popov, James Komianos, Garegin A. Papoian

**Affiliations:** 1 Department of Chemistry and Biochemistry, University of Maryland, College Park, Maryland, United States of America; 2 Institute for Physical Science and Technology, University of Maryland, College Park, Maryland, United States of America; 3 Biophysics Graduate Program, University of Maryland, College Park, Maryland, United States of America; University of Pittsburgh, UNITED STATES

## Abstract

Active matter systems, and in particular the cell cytoskeleton, exhibit complex mechanochemical dynamics that are still not well understood. While prior computational models of cytoskeletal dynamics have lead to many conceptual insights, an important niche still needs to be filled with a high-resolution structural modeling framework, which includes a minimally-complete set of cytoskeletal chemistries, stochastically treats reaction and diffusion processes in three spatial dimensions, accurately and efficiently describes mechanical deformations of the filamentous network under stresses generated by molecular motors, and deeply couples mechanics and chemistry at high spatial resolution. To address this need, we propose a novel reactive coarse-grained force field, as well as a publicly available software package, named the *Mechanochemical Dynamics of Active Networks (MEDYAN)*, for simulating active network evolution and dynamics (available at www.medyan.org). This model can be used to study the non-linear, far from equilibrium processes in active matter systems, in particular, comprised of interacting semi-flexible polymers embedded in a solution with complex reaction-diffusion processes. In this work, we applied MEDYAN to investigate a contractile actomyosin network consisting of actin filaments, alpha-actinin cross-linking proteins, and non-muscle myosin IIA mini-filaments. We found that these systems undergo a switch-like transition in simulations from a random network to ordered, bundled structures when cross-linker concentration is increased above a threshold value, inducing contraction driven by myosin II mini-filaments. Our simulations also show how myosin II mini-filaments, in tandem with cross-linkers, can produce a range of actin filament polarity distributions and alignment, which is crucially dependent on the rate of actin filament turnover and the actin filament’s resulting super-diffusive behavior in the actomyosin-cross-linker system. We discuss the biological implications of these findings for the arc formation in lamellipodium-to-lamellum architectural remodeling. Lastly, our simulations produce force-dependent accumulation of myosin II, which is thought to be responsible for their mechanosensation ability, also spontaneously generating myosin II concentration gradients in the solution phase of the simulation volume.

## Author Summary

Active matter systems have the distinct ability to convert energy from their surroundings into mechanical work, which gives rise to them having highly dynamic properties. Modeling active matter systems and capturing their complex behavior has been a great challenge in past years due to the many coupled interactions between their constituent parts, including not only distinct chemical and mechanical properties, but also feedback between them. One of the most intriguing biological active matter systems is the cell cytoskeleton, which can dynamically respond to chemical and mechanical cues to control cell structure and shape, playing a central role in many higher-order cellular processes. To model these systems and reproduce their behavior, we present a new modeling approach which combines the chemical, mechanical, and molecular transport aspects of active matter systems, all represented with equivalent complexity, while also allowing for various forms of mechanochemical feedback. This modeling approach, named MEDYAN, and software implementation is flexible so that a wide range of active matter systems can be simulated with a high level of detail, and ultimately can help to describe active matter phenomena, and in particular, the dynamics of the cell cytoskeleton. In this work, we have used MEDYAN to simulate a cytoskeletal network consisting of actin filaments, cross-linking proteins, and myosin II molecular motors. We found that these systems show rich dynamical behaviors, undergoing alignment and bundling transitions, with an emergent contractility, as the concentrations of myosin II and cross-linking proteins, as well as actin filament turnover rates, are varied.

## Introduction

The study of active matter, a far from equilibrium, self-driven system that consumes energy from the environment to generate directed motion, is at the center of many interdisciplinary fields such as physical chemistry, biophysics, and non-linear physics [1]. Active matter systems demonstrate distinctly complex dynamics and self-organization, by way of intricate energy storage and transduction, that is not observed in ordinary systems and is far from being fully understood. For example, as reviewed by Ramaswany [2], fundamentally different active matter systems, including flocks of birds and bacteria, exhibit interesting responses and order that are surprisingly similar across their varying length scales. Similarly, non-biological active matter systems, including Pt-silica particles in hydrogen peroxide solutions, become motorized and can hydrodynamically interact, displaying complex dynamics and self assembly [3, 4].

A particularly interesting example of an active matter system is the cell cytoskeleton. Being a highly dynamic polymeric network of actin filaments, microtubules, and intermediate filaments that are controlled by a diverse collection of regulatory proteins, the cytoskeleton is essential for many large-scale biological processes, including embryonic development, wound healing, and immune response [5]. The dynamic nature of the cytoskeleton allows the cell to respond to both chemical and mechanical cues, providing complex feedback mechanisms for growth and remodeling. Using molecular motors, the cytoskeleton can harness energy from ATP hydrolysis, converting it into mechanical work that can drive the system into configurations not possible with thermal motion alone. Along with the inherent nature of cytoskeletal filaments, which can assemble or disassemble rapidly due to chemical species gradients or regulatory signaling cascades, this energy consumption allows the cytoskeleton to dynamically respond to a range of extracellular stimuli on varying timescales.

Much progress has been made in recent years in modeling active networks, and in particular the cell cytoskeleton. Chemical models ranging from deterministic, ordinary differential equation as well as partial differential equation approaches describing reaction-diffusion processes [6–9], to Monte Carlo approaches that rely on spatially resolved stochastic simulation [10–13] have been used to reproduce the spatial concentration distributions and chemical dynamics of cytoskeletal networks in *in vivo* and *in vitro*. Separately, multi-scale, coarse-grained mechanical models of the cytoskeleton with limited chemical detail have been created to study its viscoelastic properties [14–16], growth and remodeling [17–19], as well as interactions with a cell membrane and surfaces [20–22]. Recently, models have been developed to investigate the active nature of cytoskeletal networks, and can reproduce many of the dynamic mechanisms involved in actomyosin contractility [23–28]. Some hybrid models have begun to incorporate multiple aspects of cytoskeletal chemistry and molecular transport with network mechanics [29–32], providing insight to the importance of this coupling in modeling and simulation.

We believe that a desirable platform for mechanochemical simulations of cytoskeletal dynamics at high structural resolution should contain the following capabilities: A) Spatially-resolved stochastic chemistry within the cytosol, the filamentous network, and between them, which would allow the establishment of global and local chemical gradients and heterogeneities, taking the fundamentally stochastic nature of chemical reactions into account. B) A sufficiently rich set of filament chemical reactions that includes (de)polymerization processes, (de)branching, formin-based nucleation and capping, monomer aging via ATP or GTP hydrolysis, severing, cross-linker and molecular motor (un)binding, and molecular motor walking, which would enable the simulation of minimally complete cytoskeletal chemistries. C) An accurate, yet computationally efficient mechanical force field, which would allow computing the deformations of a connected filamentous network that is being continuously deformed by force-generating proteins, such as myosins, as well as other chemical reaction events. D) A deep coupling between chemistry and mechanics, where, for example, the chemical heterogeneity of individual monomers in a filament due to aging leads to the corresponding spatial modulation of bending stiffness along the chain, hence, correctly localizing buckling transitions. In S1 Table, we have compiled a salient selection of current agent-based approaches for modeling cytoskeletal dynamics. To the best of our knowledge, most of the individual capabilities listed above (A-D), needed to enable next generation of structural modeling, are absent in the currently existing or prior methods [14, 16, 17, 24, 25, 27, 30–40]. Furthermore, it would be most useful to the community if the source codes for these modeling frameworks were publicly available, which is again not the case for most, but not all [30, 32], modeling frameworks listed in S1 Table. In yet another challenge, apart from the computational complexity in combining these cytoskeletal aspects, there is a need to achieve computational efficiency of scaling up simulations to micron length scales, where most interesting cytoskeletal phenomena take place, while still retaining locally high structural resolution at nanometer scale.

With the above considerations, we introduce the *Mechanochemical Dynamics of Active Networks* (*MEDYAN*) model which contains all of the aforementioned capabilities. While explicitly accounting for the complex chemical dynamics of polymers and the molecular transport of chemical species in an active network using a stochastic reaction-diffusion scheme, based on a spatially resolved Gillespie algorithm, a new coarse-grained representation and set of force fields for semi-flexible polymers has been developed, including complementary force fields for polymer branching molecules, cross-linking molecules, and molecular motors. The model also allows for mechanochemical coupling of any of these molecules, producing a full treatment of active network mechanochemistry where mechanical stresses influence chemical rate constants, allowing the modeling of Brownian ratchets, slip-bonds, catch-bonds, or more complex biphasic mechanochemical feedbacks. With this model, the complex and non-linear mechanochemical properties of active networks can be studied in full detail with efficiency, and can give insight to many active networks, including the cell cytoskeleton and other biological and artificial polymer ensembles.

Although the stochastic reaction-diffusion scheme of MEDYAN follows prior efforts from out laboratory [29, 41–45], in this work we have added significant new capabilities, including several new chemical reactions and their related mechanical elements, as well as a greatly accelerated stochastic reaction-diffusion algorithm for sparse reaction networks. But, perhaps a larger problem in cytoskeletal modeling has been the rigorous yet computationally efficient modeling of polymer mechanics in network at micron scales or above. This fundamental problem goes beyond cytoskeletal simulations and concerns many other semi-rigid polymeric melts or assembles, where there is a large discrepancy between the polymer’s persistent length and its diameter. A coarse-grained approach, based on representing polymer segments as cylinders which contain a number of monomeric units, is a natural way to address this problem. However, the difficulty is in enforcing the non-crossing constrain among the chains, where prior steric potentials were conceptually simple, but are non-analytic [16], or analytic but computationally highly inefficient in the case of large aspect ratio of polymer chain segments [46], raising serious concerns in many practical situations. In this work, we introduce a rigorous, fully analytic and computationally efficient excluded volume potential that solves this problem, enabling efficient simulations of melts of networks comprised of semi-flexible polymer chains with large aspect ratios at micron scales.

In this paper, we first introduce both the chemical reaction-diffusion and mechanical models used in MEDYAN, while also highlighting the coupling of both parts and how they work together to provide a full mechanochemical treatment of an active network. Then, to explore the capabilities of this model and its publicly available software implementation (available at www.medyan.org), we investigate a contractile actomyosin network containing actin filaments, *α*-actinin cross-linking proteins, and non-muscle myosin IIA mini-filaments, demonstrating the propensity for rich dynamical remodeling of these networks, as their mechanochemistry is tuned by varying myosin II and cross-linker concentrations. Our simulations indicate a clear threshold of cross-linker concentration which induces contractile behavior of actin filaments by myosin II mini-filaments in a smaller 1 × 1 × 1 *μm* ^3^ actomyosin system, as well as other distinct network morphology changes. In particular, our analyses clearly indicate that in all simulated systems actin filaments tend not only geometrically align, but, surprisingly, this alignment is unipolar (emerging from an initially random, disordered network). We further found that both this polarity alignment and contractile behavior are tightly regulated by the extent of actin filament turnover, producing biphasic super-diffusive motions of individual actin fibers driven by myosin II mini-filament force generating activity. We also discuss myosin II mini-filament force-dependent accumulation in these systems, as all simulated concentration configurations and system sizes produce this accumulation in areas of high network stress, spontaneously generating concentration gradients in the solution phase. In a larger 3 × 3 × 3 *μm* ^3^ actomyosin system, we observed a distinct alignment, contraction and polarity sorting, reminiscent of arc formation in the rear of a lamellipodium.

## Models

### Chemical model

The cell cytoskeleton, as well as other active networks, takes advantage of distinct chemical phenomena which allows the network to grow and remodel based on extracellular signaling and other chemical cues [47, 48]. In order to model the complex chemical interactions that occur in these dynamic networks at a microscopic resolution, the MEDYAN model uses a stochastic reaction-diffusion scheme based on a three dimensional, spatially resolved Gillespie algorithm [49, 50] as in previous works [29, 41–45]. With simulation space divided into compartments, with compartment size chosen based on the so-called “Kuramoto length” of the reaction-diffusion system of interest [51, 52] (see Section B of S2 Text for an example determination of a Kuramoto length), diffusion and other transport events of chemical species, which could include active transport via molecular motors or convective transport such as retrograde flow, are modeled as stochastic jumps between compartments that can be directionally biased or unbiased in order to model various transport mechanisms. This allows for a discrete and spatially resolved treatment of small copy numbers and non-uniform concentration gradients, which could produce substantial and important fluctuations in chemical dynamics at the nanoscale. In particular, recent works have studied the significant effects of these stochastic fluctuations on filopodial growth [41, 45] as well as the effects of active transport phenomena and its significance in both lamellipodia and filopodia formation and sustainability [29, 43, 44]. In these systems, the concentration of G-actin monomers as well as other cytosolic molecules fluctuates greatly across the spatial domain of the protrusion due to both diffusion and active transport mechanisms, producing non-linear chemical response and signaling. These important effects could not be captured with deterministic approaches, which ignore the cytoskeleton’s biologically inherent stochasticity.

In the MEDYAN model, we have developed the stochastic reaction-diffusion scheme further such that one can use varying types of stochastic simulation algorithms in order to optimize a simulation based on the chemical properties of the simulated network. While the original Gillespie algorithm is an efficient and exact alternative to solving a chemical master equation [49, 50], with the chemical master equation being nearly impossible to solve for the complexity of systems we are considering, optimized methods have been developed for the original Gillespie *direct method* to decrease computational complexity for loosely-coupled chemical reaction networks, as reviewed by Cao et al. [53]. In particular, the *next reaction method*, developed by Gibson and Bruck [54], makes use of clever data structures to optimize the propensity updating process after each reaction is executed, producing massive speed-ups for sparse reaction-diffusion networks compared to the original algorithm. The MEDYAN model can make use of either of these algorithms depending on the type of chemical system to be simulated. In most cases, the latter is more suitable for simulating most active networks, where the chemical reactions across the system are sparse and spatially localized by compartments. With these algorithm optimizations, the computational complexity for stochastically simulating active network evolution is greatly reduced, allowing the model to surpass timescales accessible with the original Gillespie schemes. The MEDYAN software implementation, which is discussed in Section D of S1 Text, is also designed such that such that new stochastic simulation algorithms can easily be included in the existing reaction-diffusion framework, including the *optimized direct method* [53] and *partial propensity methods* [55, 56]. For a detailed benchmarking of the currently implemented optimizations in systems similar to the ones simulated in the Results section, see Section A of S4 Text.

In order to account for the chemical heterogeneity of active network polymers, we represent them in the model as a distinct arrangement of chemical monomers that are overlayed onto the existing reaction-diffusion compartment grid, which allows them to undergo spatially resolved reactions with diffusing chemical species besides typical polymerization and depolymerization events. This can be of importance to network dynamics in the case of actin filaments, where polymerized actin hydrolyzes ATP, giving rise to a substantial change in polymerization kinetics at both ends of the filament [57, 58]. In conjunction with hydrolysis, the cytoskeletal regulatory protein ADF/Cofilin can sever actin filaments preferentially where ATP has been hydrolyzed [59, 60]. Together, and along with other chemical interactions in the cytoskeleton, these reactions are responsible for the actin filament turnover process observed in most types of cellular protrusions [61]. With the MEDYAN polymer representation, these important molecular processes can be included in the reaction-diffusion master equation (RDME) and simulated in full detail.

We have also included detailed cross-linker chemical dynamics to the model. It has been well known that cross-linking molecules are important for producing the observed morphology of the actin cytoskeleton *in vivo* [62, 63], but most existing cytoskeletal models do not include the stochastic binding and unbinding of cross-linkers to actin filaments in the simulation space. In the MEDYAN model, cross-linker binding reactions with neighboring polymers are dynamically added; if two separate polymer binding sites are within a specified range in a given compartment, an unbound cross-linker species in that compartment can bind to them. An unbinding reaction is also associated with that molecule once bound, which can then release it from both polymers. This dynamic addition of reactions allows for computational efficiency as well as an exact, spatially resolved treatment of cross-linking molecules, which can be essential for active network evolution. See the Mechanical model section for a more detailed description of the mechanical interactions of cross-linking molecules.

In order to make a simulated network active, we have introduced molecular motors in the model—molecules which utilize energy released due to chemical reactions in the system and transfer it into mechanical work. For example, in cytoskeletal networks, energy from ATP hydrolysis is used by number of protein species to generate forces. In particular, the non-muscle myosin II (NMII) motor family plays a significant role in cytoskeletal remodeling and cell motility [64, 65], where individual NMII motors assemble into larger bipolar filaments that can reach hundreds of nanometers in length [66]. The MEDYAN model can include bipolar NMII filaments that, in a similar manner to cross-linking molecules, can bind onto two neighboring actin filaments. The slow diffusion of these larger molecules may produce some spatial diffusion error on a compartment grid, and hybrid combinations of Brownian dynamics and stochastic reaction-diffusion models have been introduced in recent years [67] as a way to solve this error, which could be included in the MEDYAN model in the future. But, we believe for grids used in the Results section which are 500 *nm* in length, this is still a good estimate of true diffusive behavior. When bound, the head ensembles can make stochastic directional steps towards the barbed end of either filament, which generates “sliding” forces in the network, promoting reorganization and contractility. See the Mechanical model section for a more detailed description of the mechanical interactions of NMII filaments.

**Fig 1:**
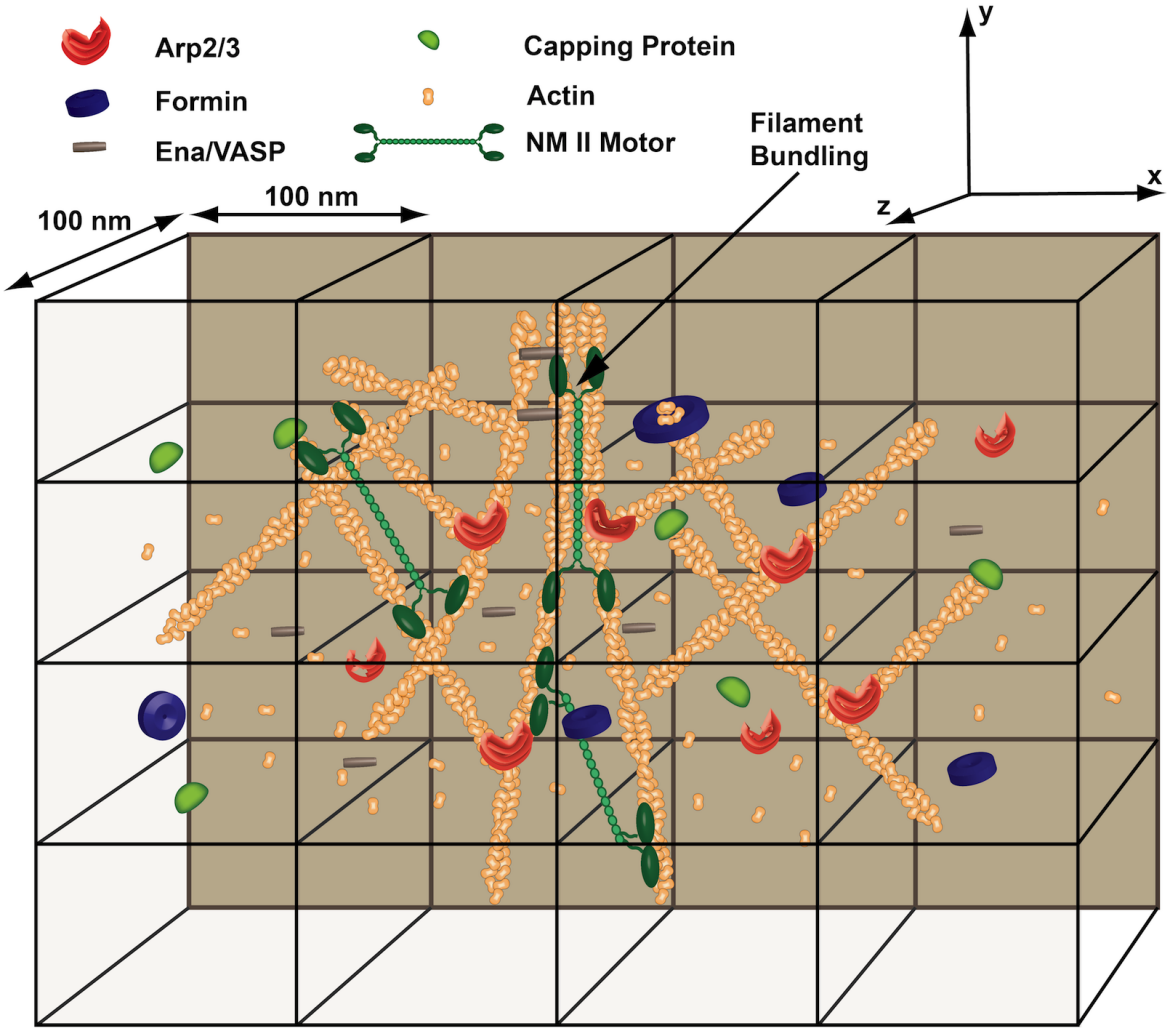
A cytoskeletal network in the MEDYAN model. A complex cytoskeletal network can be simulated with MEDYAN’s stochastic reaction-diffusion scheme. Chemical interactions will cause complex network evolution, such as the process of actin filament bundling. See Section A in S1 Text for a more detailed description of all chemical reactions that can be included in a simulation.

In a MEDYAN simulation, a transport event or polymer-related reaction is chosen to occur by the stochastic simulation algorithm based on its reaction propensity. This process repeats, advancing the chemical reaction-diffusion system in time. Bulk reactions can also be included between diffusing species, allowing for even more complex chemical evolution. See Section A in S1 Text for a more detailed description of the entire set of chemical reactions that can be simulated. With the stochastic reaction-diffusion scheme and polymer representation described, complex active networks can be simulated with explicit and detailed chemical interactions and molecular transport. Fig 1 shows a cartoon depiction of a cytoskeletal network that could be simulated with the MEDYAN model. All molecules can diffuse throughout the simulation space according to their specified diffusion rate and the chosen compartment size. Actin filaments can grow and shrink due to the polymerization and depolymerization of G-actin monomers, as well as the binding and unbinding of capping proteins and formins, and Arp2/3 can nucleate new actin filaments on existing filaments at a 70° angle [68]. Lastly, cross-linking proteins can bind and unbind to actin filaments, and NMII mini-filaments can bind, unbind, and walk along actin filaments.

### Mechanical model

To complement the detailed stochastic reaction-diffusion scheme described above, we have developed a new set of force fields in the MEDYAN model to account for the mechanical properties of an active network. In previous work [29, 42], a simple bead-spring model was used to describe actin filament mechanics, where a single filament was regarded as a composition of hard-core beads. These beads represented individual monomers which were then connected by either a harmonic or more complex potential. This method, while being a detailed and robust description, required the calculation of a large number of interactions between neighboring beads during a mechanical equilibration of the system. Considering that a cubic micron of a cytoskeletal network could contain on the order of 10^6^ actin monomers, mechanical equilibration of a system with this simple model would severely limit simulation timescales that could be accessed.

**Fig 2:**
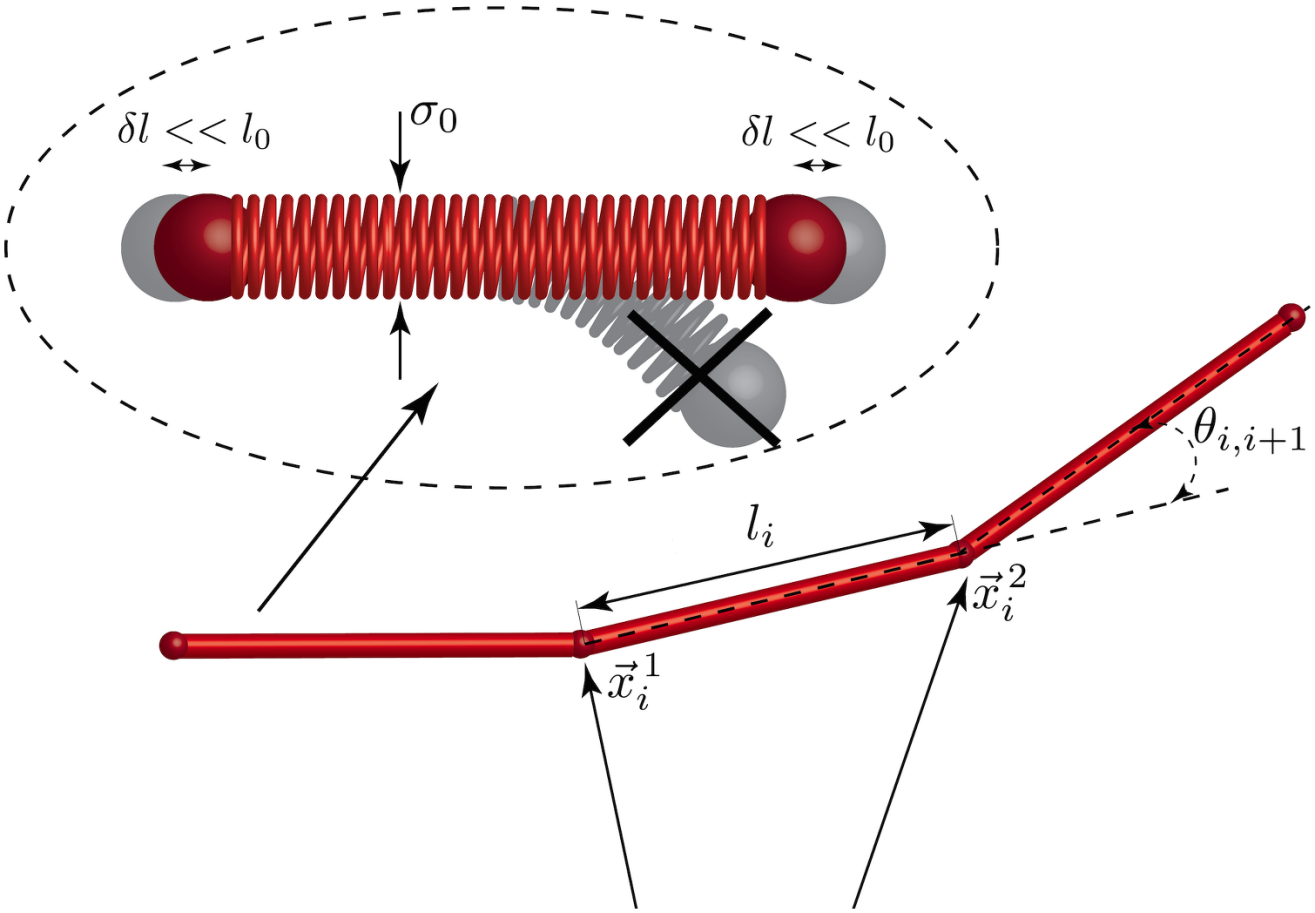
A polymer in the MEDYAN model. A cylinder based scheme is used in the MEDYAN force fields to model semi-flexible polymers. Here, *σ*_0_ is the diameter of the cylinder and *l*_0_ is the equilibrium length, where *l*_0_ > > *σ*_0_. We assume that axial deformations of the cylinders are small and radial deformations are forbidden.

In order to overcome these computational limitations, we are introducing in this work a polymer model based on elongated cylindrical monomer segments for simulating semi-flexible polymers with a persistence length, denoted as *l*_*p*_, that is much larger than its diameter *σ*_0_ (i.e. very large aspect ratio, *l*_*p*_ > >*σ*_0_). Cylinders have been previously introduced in various coarse-grained computational models for the description of systems containing elongated objects, including the modeling of viscoelastic actin networks [16] and hydrodynamics of suspensions [69, 70]. Here we would like to emphasize that cylinders in the MEDYAN description are not considered as collections of beads, but rather as stiff weightless springs of diameter *σ*_0_, connecting its end points. This fact, as it will be seen later, will help us to build up a rather intuitive mathematical formalism to describe polymer mechanics. Fig 2 represents the scheme of using cylinders as monomer units in a polymer chain. This assumption makes the model applicable for the description of most biopolymers (in the case of actin filaments, *l*_*p*_/*σ*_0_ ≈ 10^3^), and while force-generating molecular motors could significantly change the correlation between two points along the polymer chain, these correlation lengths will still be significantly larger than the distance between two neighboring monomers in previously used bead-spring model. Moreover, the new model can describe flexible molecules as well, as a standard bead-spring model can be considered as a limit with *l*_*p*_ → *σ*_0_. For a detailed benchmarking of this coarse-graining scheme in systems similar to the ones simulated in the Results section, see Section B of S4 Text.

We now introduce the interaction potentials used in the MEDYAN model. We note that the MEDYAN software implementation can easily be modified to include different types of potentials for the interactions presented below; see Section D of S1 Text for a more detailed discussion on the implemented software’s flexibility for the addition of various interaction potentials. For example, a finitely extensible nonlinear elastic (FENE) potential could be easily added to the existing code for less elastic semi-flexible polymers, molecular motors, and cross-linkers [71]. Other forms of polymer excluded volume effects could also be included.

We assume that every coarse-grained monomer segment is represented by a cylinder with a finite thickness *σ*_0_ and equilibrium length *l*_0_, as shown in Fig 2. To account for filament bending, we use an angular potential between consecutive cylinders in the polymer chain, written as

$$U_{i}^{\text{bend}}=\varepsilon_{\text{bend}}\left[1-\cos\left(\theta_{i,i+1}\right)\right],$$ (1)

where *θ*_*i*, *i*+1_ is the angle between two consecutive cylinders *i* and *i* + 1 along the polymer chain, and *ε*_bend_ is the bending energy, which can be chosen based on the persistence length of the simulated polymer.

Cylinders also can be slightly stretched or compressed along their main axis, while radial deformations within the cylinder are not allowed. To illustrate this fact we draw springs inside of the cylinders in Fig 2. The stretching energy corresponding to deformations of the *i* ^th^ cylinder can be represented as

$$U_{i}^{\text{str}}=\frac{1}{2}K_{\text{str}}{\left(|{\vec{l}}_{i}|-l_{0}\right)}^{2},$$ (2)

where ${\vec{l}}_{i}={\vec{x}}_{i}^{\;2}-{\vec{x}}_{i}^{\;1}$ is the vector connecting the endpoints of the *i* ^th^ cylinder, and *K*_str_ is the stretching constant. As in the bending potential, this constant, along with the equilibrium length, *l*_0_, can be chosen depending on the elastic modulus of the simulated polymer. In the case of actin filaments, these bending and stretching potentials allow the model to capture non-linear deformations reported by various studies [72–74]; with $U_{i}^{\text{bend}}$ accounting for the thermal elasticity of the chain, $U_{i}^{\text{str}}$ describes elastic deformations of the chain stretched beyond its entropically driven elastic limit [75]. These deformations are considered to have high energy penalties, which is reflected in high values of *K*_str_, therefore, can occur only under very large global deformations of the system.

There are several common approaches usually used to calculate excluded volume interactions between two aspherical elongated particles, which are cylinders in our case. The most obvious approach is to represent the elongated particles as a collection of spheres; with this representation, interactions are simply calculated as a sum of pairwise hardcore repulsions between the spheres forming each cylinder. While this is a very simple and straightforward method, it defeats all purpose and efficiency of the initial cylindrical coarse-graining. Another widely used approach is to use the Gay-Berne potential to describe excluded volume interactions between interacting cylinders [46, 76], which can be used as a part of the LAMMPS [77] package. This potential, however, has limited applicability and lower computational efficiency when *l*_*p*_ > > *σ*_0_ as in the case of most biopolymers. On top of that, computational complexity of this potential is also increased greatly due to constantly finding the distance of closest approach between the two cylinders, which is a very costly calculation. Finally, another method was used in the model of Kim et al. [16] which calculates cylindrical repulsive interactions using the closest distance between two interacting segments. This force is then transferred to the end points of the segments, based on the lever rule as well as the position of the point of closest approach. Despite the elegance of this method, we found several drawbacks for using this approach in the MEDYAN model: from a computational point of view, algorithms for calculating the point and the distance of closest approach between neighboring cylinders contains costly control flow as mentioned previously, increasing computational complexity for this approach greatly. From a mathematical and physical point of view, a lack of a continuous and analytical function for this closest distance puts limitations on the resulting force calculations, which might lead to oscillations and divergence during mechanical equilibration of the system.

In order to overcome these issues, we introduce a novel approach for calculating excluded volume interactions between two cylinders. This approach is conceptually similar to early mentioned devision the cylinders into small point-like subunits and calculating interactions between them. However, instead of an actual representation of the cylinders as a collection of subparticles, we solve this analytically by writing a pair potential between two infinitely small fragments on both cylinders and then integrating this pair potential over the length of both cylinders.

The potential of excluded volume interactions between two cylindrical units on neighboring polymers, denoted as *i* and *j*, can be given by:

$$U_{ij}^{\text{vol}}=\underset{l_{i},l_{j}}{\;\int \int \;}\delta U(|{\vec{r}}_{i}-{\vec{r}}_{j}\left|\right)dl_{i}dl_{j}.$$ (3)

**Fig 3:**
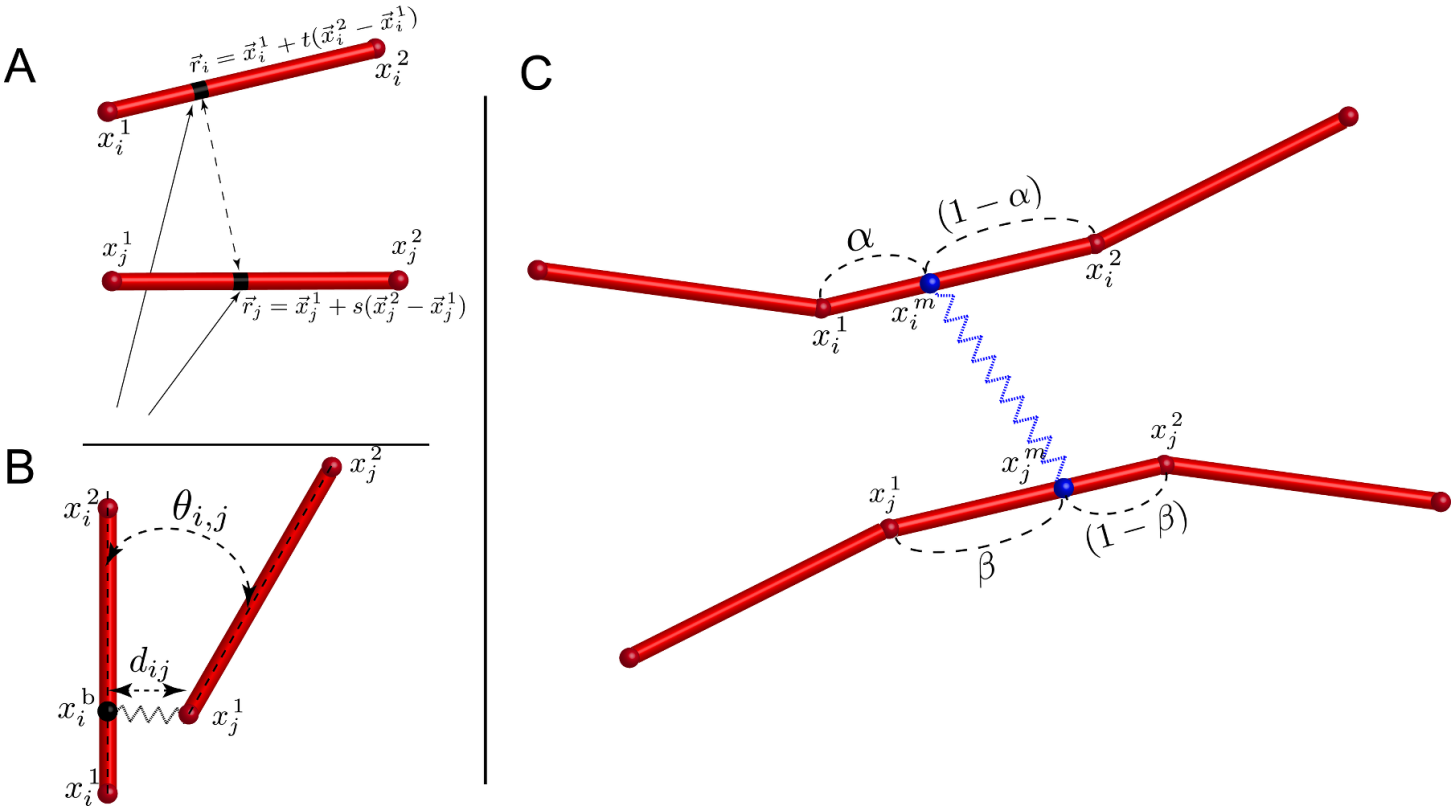
Interactions in the the MEDYAN model. A) A Schematic representation of two arbitrary points on the cylinders *i* and *j*, used to calculate excluded volume interactions. B) Representation of branching points. Position of the branching point ${\vec{x}}_{i}^{\;\text{b}}$ on the “mother” filament is determined by a stochastic chemical reaction. C) Representation of the motor as a potential between two points ${\vec{x}}_{i}^{\;\text{m}}$ and ${\vec{x}}_{j}^{\;\text{m}}$ on bound cylinders. Positions of the binding points *α* and *β* are determined by stochastic chemical reactions.

Here, $\delta U(|{\vec{r}}_{i}-{\vec{r}}_{j}|)$ is the above mentioned pair potential between two points located on the two interacting cylinders *i* and *j* as shown in Fig 3A. For pure excluded volume repulsion, we have chosen $\delta U(|{\vec{r}}_{i}-{\vec{r}}_{j}\left|)=1/\right|{\vec{r}}_{i}-{\vec{r}}_{j}{|}^{4}$. This provides a steep enough function to mimic cylindrical hard core repulsion, while allowing the integrals in Eq 3 to be evaluated analytically. This allows us to derive analytical expression for the forces acting on the end points of the cylinders *i* and *j*. For every arbitrary point on a given cylinder *i* we can write the parametric equation ${\vec{r}}_{i}={\vec{x}}_{i}^{\;1}+t\left({\vec{x}}_{i}^{\;2}-{\vec{x}}_{i}^{\;1}\right)$, where ${\vec{x}}_{i}^{\;1}$ and ${\vec{x}}_{i}^{\;2}$ are coordinates of the beginning and the end of cylinder *i*, respectively, and *t* ∈ [0, 1] is a parameter. Taking this into account, and writing a similar parametric equation for cylinder *j*, Eq 3 can be written as

$$U_{ij}^{\text{vol}}=K_{\text{vol}}\int_{0}^{1}\int_{0}^{1}\frac{dsdt}{|{\vec{r}}_{i}\left({\vec{x}}_{i}^{\;1},{\vec{x}}_{i}^{\;2},t\right)-{\vec{r}}_{j}\left({\vec{x}}_{j}^{\;1},{\vec{x}}_{j}^{\;2},s\right){|}^{4}},$$ (4)

where *K*_vol_ is a constant determining the strength of repulsion.

The MEDYAN model accounts for the process of polymer nucleation by branching. See the Chemical model section for a more detailed description of branching nucleation events. We introduce the following potential to describe the mechanical interactions of branched polymers, as seen in Fig 3B:

$$U_{ij}^{\text{branch}}=U_{ij}^{\text{branch,str}}+U_{ij}^{\text{branch,ang}}+U_{ij}^{\text{branch,dihed}},$$ (5)

where this interaction regards cylinder *i* as being on the “mother” polymer and cylinder *j* as the “daughter” or branched polymer.

The first term in Eq 5, which is a potential securing cylinder *j* to a branching point on cylinder *i*, can be written as

$$U_{ij}^{\text{branch,}\;\text{str}}=K_{\text{branch,str}}{\left(|d_{ij}|-d_{0}\right)}^{2},$$ (6)

where ${\vec{d}}_{ij}={\vec{x}}_{j}^{\;1}-{\vec{x}}_{i}^{\;\text{b}}$ is the distance between branching point on the cylinder *i*, ${\vec{x}}_{i}^{\;\text{b}}$, and the end point of the cylinder *j*, ${\vec{x}}_{j}^{\;1}$, *d*_0_ is the equilibrium value for this distance, and *K*_branch, str_ is the stretching constant that can be chosen depending on the stiffness of the simulated branching molecule. As it was noted previously, we assume that axial deformation of the cylinders are small, and radial deformations are prohibited. In this case we can describe position of any branching point on the cylinder in terms of a scalar value *γ* ∈ [0, 1], which represents a fractional position of the branching point ${\vec{x}}_{i}^{\;\text{b}}$ with respect to end points of the cylinder ${\vec{x}}_{i}^{\;1}$ and ${\vec{x}}_{i}^{\;2}$, ${\vec{x}}_{i}^{\;\text{b}}=\left(1-\gamma \right){\vec{x}}_{i}^{\;1}+\gamma {\vec{x}}_{i}^{\;2}$. In other words, *γ* will be generated as the result of a chemical branching event and will not depend on the stress generated in the branching junction; see the Chemical model section for more details on this chemical event and its effects.

The second term in Eq 5 describes an angular potential at the chosen branching point between cylinders *i* and *j*:

$$U_{ij}^{\text{branch,ang}}=\varepsilon_{\text{branch,ang}}\left[1-\cos\left(\theta_{i,j}-\theta_{0}\right)\right],$$ (7)

where *θ*_0_ is the equilibrium value of the branching angle, *θ*_*i*, *j*_ is the angle between cylinders *i* and *j*, and *ε*_branch,ang_ is the angular bending energy, which can be chosen based on the flexural rigidity of the branching molecule. In case of actin filaments, Arp2/3 grows nucleated filaments at an equilibrium angle *θ*_0_ ≈ 70° to the mother filament [68].

Finally, the last term in Eq 5 describes a dihedral potential between cylinders *i* and *j*, which uses the dihedral angle between two planes, formed by the points $\left(x_{i}^{\;2},x_{i}^{\;b},x_{j}^{\;1}\right)$ and $\left(x_{i}^{\;b},x_{j}^{\;1},x_{j}^{\;2}\right)$:

$$U_{ij}^{\text{branch,dihed}}=\varepsilon_{\text{branch,dihed}}\left[1-\cos\left(\phi_{i,j}-\phi_{0}\right)\right],$$ (8)

where

$$\phi_{i,j}=\arccos\left({\vec{n}}_{i}\cdot {\vec{n}}_{j}\right),\;{\vec{n}}_{i}=\frac{\left[\left({\vec{x}}_{i}^{\;2}-{\vec{x}}_{i}^{\;\text{b}}\right)\times {\vec{d}}_{ij}\right]}{|\left({\vec{x}}_{i}^{\;2}-{\vec{x}}_{i}^{\;\text{b}}\right)\left|\;\right|{\vec{d}}_{ij}|},\;\text{and}\;{\vec{n}}_{j}=\frac{\left[{\vec{l}}_{j}\times {\vec{d}}_{ij}\right]}{|{\vec{l}}_{j}\left|\;\right|{\vec{d}}_{ij}|}.$$

The symbols (⋅) and [×] stand for scalar and vector product, respectively, and *ε*_branch,dihed_ represents the dihedral bending energy between the two cylinders, which can be chosen in a similar manner to *ε*_branch,ang_.

The MEDYAN model incorporates molecular motors into the network as a dynamic object which can bind onto neighboring cylinders *i* and *j* at the positions ${\vec{x}}_{i}^{\;m}$ and ${\vec{x}}_{j}^{\;m}$, and create a mechanical bond, as shown in Fig 3C. See the Chemical model section for more description of motor binding and walking events. For computational efficiency in studies of global deformations in large active networks under the force generation of small molecular motor ensembles, an implicit representation for molecular motors has been developed in which the motor is represented as a single potential acting on two neighboring cylinders. In the case of modeling myosin II mini-filaments, which are small ensembles of 10–30 myosin heads aligned in a bipolar fashion [78], this is an excellent approximation. In future studies, a more explicit implementation of molecular motors, comprised of connected monomer units, can be implemented to allow a more detailed and accurate description of myosin II filaments at the cost of computational efficiency. This explicit representation may be of importance in studies including myosin II thick filaments, which can contain on the order of 100–800 motor heads [79], thus allowing the ensemble to bind to a large number of actin filaments simultaneously.

To describe the stretching energy of a bond created by an implicit motor, we introduce the following harmonic potential:

$$U_{ij}^{\text{motor}}=\frac{1}{2}K_{\text{motor}}{\left(|{\vec{l}}_{ij}^{\;\text{m}}|-l_{0}^{\text{m}}\right)}^{2},$$ (9)

where ${\vec{l}}_{ij}^{\;\text{m}}={\vec{x}}_{j}^{\;\text{m}}-{\vec{x}}_{i}^{\;\text{m}}$ is the instantaneous length of the motor, $l_{0}^{m}$ is the equilibrium length of the particular motor, and *K*_motor_ is the stretching constant, which can be chosen based on the stiffness of molecular motor to be simulated.

The binding position of the motor head ${\vec{x}}_{i}^{\;\text{m}}$ on cylinder *i* can be expressed as ${\vec{x}}_{i}^{\;\text{m}}=\left(1-\alpha \right){\vec{x}}_{i}^{\;1}+\alpha {\vec{x}}_{i}^{\;2}$ where *α* ∈ [0, 1]. Here, similar to the case of the branching potential in Eq 6, we assume that *α* is a scalar parameter, which does not change during mechanical minimization and is determined by a stochastic chemical event. Using this representation along with a similar expression for the binding position on cylinder *j*, we can write ${\vec{l}}_{ij}^{\;\text{m}}$ as

$${\vec{l}}_{ij}^{\;\text{m}}=\left(1-\beta \right){\vec{x}}_{j}^{\;1}+\beta {\vec{x}}_{j}^{\;2}-\left(1-\alpha \right){\vec{x}}_{i}^{\;1}-\alpha {\vec{x}}_{i}^{\;2}.$$ (10)

where *β* ∈ [0, 1] represents the fractional position on cylinder *j*. As the result of chemical reactions, *α* and *β* can stochastically change, which results in motor head relocation and the generation of new mechanical stresses in the system.

Similarly, passive cross-linkers are represented using the potential in Eq 9, but with time-independent values of *α* and *β*. Again, see the Chemical model section for more description of cross-linker binding and unbinding events. In a similar manner to molecular motors, by not explicitly introducing new classes of interactions for these molecules, but instead using analytically computed energies and forces between neighboring cylinders connected by passive cross-linkers (i.e. relying on an implicit mechanical representation), the MEDYAN model can achieve much higher computational efficiency in the simulation of large active networks with these molecules.

System boundaries in MEDYAN are modeled as non-deformable shells with a number of possible shapes, including cubic, spherical, and capsule geometries. These boundaries sterically repel approaching polymer segments, keeping the simulated network confined in the chosen domain. One of the possible potentials used to describe the interaction between the *i* ^th^ cylinder and the boundary can be written as:

$$U_{i}^{\text{boundary}}=\varepsilon_{\text{boundary}}\;e^{-\vec{d_{i}}/\lambda },$$ (11)

where *λ* is the screening length and $\vec{d_{i}}$ is the distance between the boundary and the closest endpoint of the *i* ^th^ cylinder, ${\vec{x}}_{i}^{\;2}$ or ${\vec{x}}_{i}^{\;1}$. *ε*_boundary_ represents the repulsive energy provided by the boundary.

The total energy of the system *U* ^tot^, assuming all corresponding species were chemically generated, is equal to a sum of the above contributions. This energy is then used in the MEDYAN model to mechanically equilibrate the system after a number of stochastic chemical reaction-diffusion steps. In order to perform this equilibration efficiently, most methods require analytical expressions for the derivatives of the energy with respect to cylinder position, *e.g.* forces in Langevin dynamics or gradient directions in conjugate gradient methods. Note that all terms in *U* ^tot^ (Eqs 1–11) but Eqs 6 to 9 are initially written in terms of the end points of the cylinders; so, derivatives of those terms can be taken with respect to ${\vec{x}}^{\;1}$ and ${\vec{x}}^{\;2}$ such that, if using “force” terminology, will give forces acting on these end points of the cylinders. Eqs 6 to 9 also include coordinates of points located on the cylinders somewhere in between its end points: branching position on the “mother” filament in Eqs 6 to 8 and motor or cross-linker head positions in Eq 9. However, as it was discussed before, for every point *m* along cylinder *i* we can write ${\vec{x}}_{i}^{\;\text{m}}=\left(1-\alpha \right){\vec{x}}_{i}^{\;1}+\alpha {\vec{x}}_{i}^{\;2}$, where *α* ∈ [0, 1] does not depend on the coordinates of the cylinder end points or stresses in the system during during a mechanical equilibration (see Mechanochemical coupling). Taking this into account, Eqs 6 to 9 can be rewritten only in terms of positions of cylinder end points. Therefore, these potentials can be differentiated with respect to only ${\vec{x}}^{\;1}$ and ${\vec{x}}^{\;2}$. This assumption follows under the condition of small axial deformations of the cylinders and the absence of radial deformations within each cylinder (see Fig 2), appropriate for relatively stiff filaments, such as F-actin and many other biological and artificial polymers. Very soft polymers, on the other hand, would be more profitably modeled as comprising of spherical beads and not cylinders. Mathematically speaking, this is equivalent to a simple chain rule:

$$\frac{\partial U^{i}}{\partial {\vec{x}}_{i}^{\;\text{m}}}=\left(1-\alpha \right)\frac{\partial U^{i}}{\partial {\vec{x}}_{i}^{\;1}}+\alpha \frac{\partial U^{i}}{\partial {\vec{x}}_{i}^{\;2}}.$$

From a mechanical point of view, this is equivalent to transferring of a force applied at a point ${\vec{x}}_{i}^{\;\text{m}}$ to cylinder end points according to a lever rule, which was also used in [16]. Hence, to compute instantaneous forces needed for mechanical energy minimization in a system with the interaction potentials introduced in this section, we only need to calculate

$$\frac{\partial U^{\text{tot}}}{\partial {\vec{x}}_{i}^{\;1}},\;\text{and}\;\frac{\partial U^{\text{tot}}}{\partial {\vec{x}}_{i}^{\;2}},$$ (12)

where the notation $\frac{\partial }{\partial \vec{x}}=\left\{\frac{\partial }{\partial x_{x}},\frac{\partial }{\partial x_{y}},\frac{\partial }{\partial x_{z}}\right\}$ represents the gradient in the direction of $\vec{x}$. This formalism allows us to calculate not only point-like interactions that can be described by a lever rule, but also more complex interactions, where the level cannot be applied, as in the case of our newly introduced cylindrical excluded volume potential (Eq 3). With these forces, an energy minimization is performed using a conjugate gradient method in the current MEDYAN software implementation, and is designed such that optimized minimization methods can be easily added to the existing code; see Section D of S1 Text for more description.

### Mechanochemical coupling

**Fig 4:**
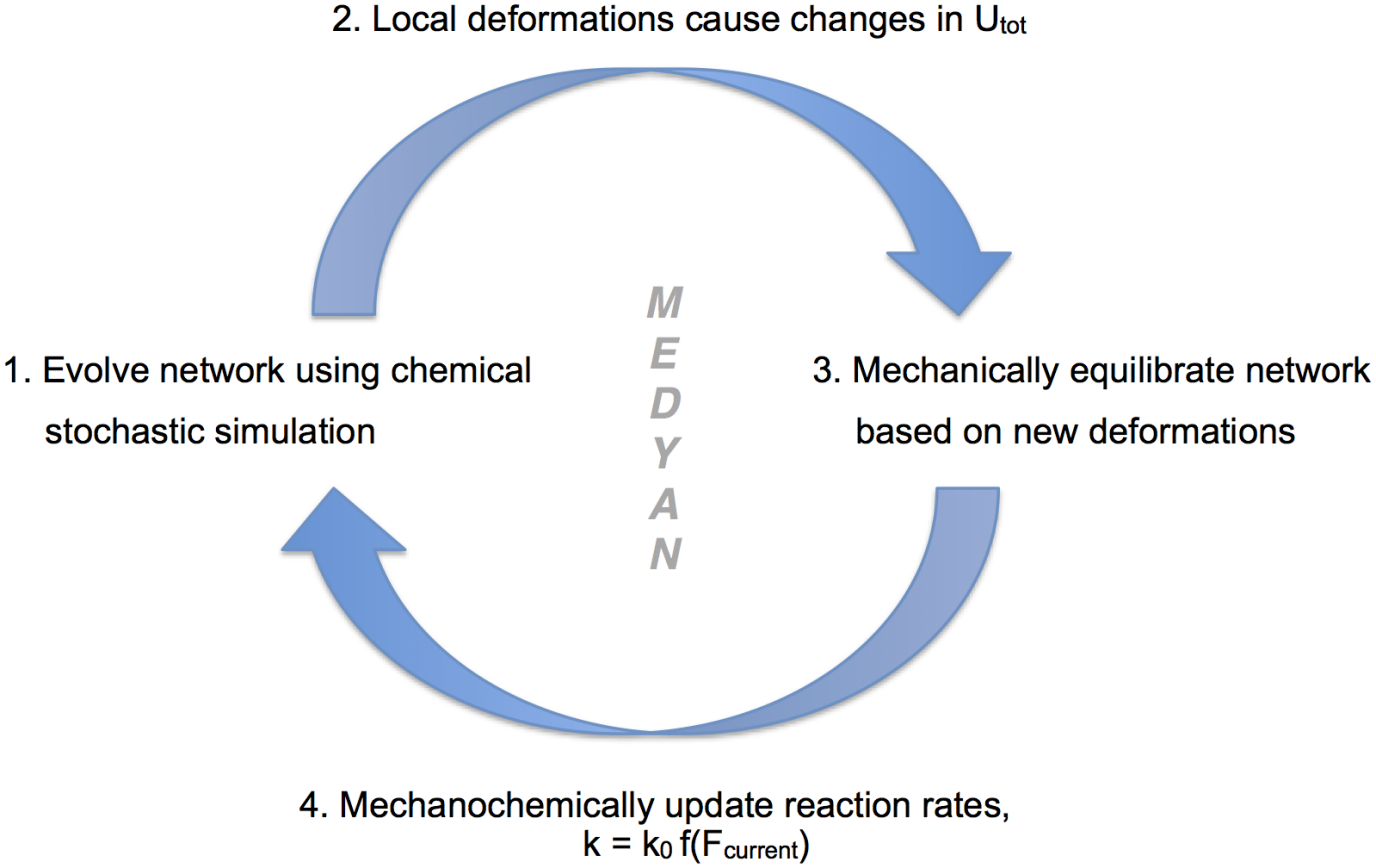
A flow diagram of a MEDYAN simulation. (1) After the chemical stochastic simulation evolves the network in time and (2) local deformations are formed, (3) a mechanical equilibration is performed and (4) reaction rates are updated according to chosen functional forms *f*(*F*_*current*_) where *F*_*current*_ is the force on that reacting molecule after equilibration.

In a MEDYAN simulation, the chemical and mechanical models work in tandem to evolve an active network in time. Fig 4 shows the general flow of the entire MEDYAN trajectory, where timescale separation of slower chemically-driven force generation and faster local force relaxation in a simulated active network allows for an iterative switching between stochastic chemical simulation and mechanical equilibration. After the stochastic simulation algorithm executes a set number of chemical steps to evolve the network in time, some of which have mechanical effects that drive the network slightly out of mechanical equilibrium, the energy of the network will be minimized according to the force fields specified in the simulation. For a detailed description of mechanical equilibration, as well as a list of mechanic effects of various chemical reactions, see Sections B and C in S1 Text. By performing highly efficient chemical stochastic simulation coupled with coarse-grained semi-flexible polymer chain mechanics, active network simulations with the MEDYAN model can reach time and length scales not accessible by its preceding models [29, 42] with this high level of resolution in both aspects of stochastic reaction-diffusion and coarse-grained polymer chain mechanics; see Section C of S4 Text for a note on time and length scales attainable with MEDYAN compared to previous work.

The above-stated iterative simulation protocol assumes that the mechanical subsystem is always near equilibrium, adiabatically following the slow chemical dynamics at all incremental time points during a simulation of an active network evolution. This is a valid approximation in the case of typical actin cytoskeletal networks undergoing small, localized force deformations, as evidenced by the recent microrheology experiment of Falzone et al. [80]. Their measurements of the relaxation time of various mesh-sized deformations in an actin filament network indicated an upper limit of approximately 10 milliseconds, which is significantly faster compared to the the walking rate of non-muscle myosin II motors [81] or actin filament growth rates [57] under physiological concentrations. While this timescale separation holds for most cytoskeletal networks undergoing typical molecular motor or filament growth-induced deformations, ones with slower network stress relaxation, possibly due to larger-scale network deformations or very fast reaction-diffusion processes, or if thermal motions need to be studied for other reasons, may be better served with a Langevin thermal dynamics approach at the cost of significantly reduced computational efficiency. In the latter case, the mechanical subsystem will evolve under constant time step Langevin dynamics, where it may be then more convenient to evolve the reaction-diffusion subsystem employing one of the multiparticle RDME methods [82, 83] instead of a variable time-step algorithm such as the next subvolume approach used in the current work.

The MEDYAN model also allows for the explicit coupling of both separate chemical and mechanical entities such that one can simulate the mechanochemical feedbacks of an active network. Many molecules in active networks, and in particular the cell cytoskeleton, have distinct mechanochemical properties that can greatly affect overall network dynamics and morphology [84–86]. MEDYAN allows for a detailed treatment of these relationships by dynamically updating reaction rates based on a reacting molecule’s evolving stresses, and any form of mechanochemical effect can be included in the model. Once the system is mechanically equilibrated following a number of chemical steps, reaction rates are updated based on newly formed mechanical deformations as shown in Fig 4. With chemical, mechanical, and molecular transport properties of an active network being treated on equal footing, as well as their coupling being explicitly accounted for, the MEDYAN model allows simulations of various active networks with great mechanochemical detail and efficiency.

### Publicly available software package

The MEDYAN model has been implemented in a C++ software package which uses efficient data structures and object-oriented programming paradigms to simulate active networks with the scheme described in the earlier sections. The package has the capability for the user to specify the geometric, chemical, and mechanical properties of the simulated active network in a number of system input files, making the code robust and flexible enough to perform simulations for a range of active matter systems. This package, along with documentation on usage and compilation, as well as a visualization tool, is publicly available for use, modification, and addition of new patches (www.medyan.org). See Section D of S1 Text for a more detailed description of the software implementation.

## Results

We relied on the the capabilities of the MEDYAN model to investigate the time evolution and contractility of an actomyosin network comprised of actin filaments, non-muscle myosin IIA (NMIIA) mini-filaments, and *α*-actinin cross-linkers. Actomyosin contractility is a mechanochemical phenomenon that is essential in a cell’s response to extracellular environments [85, 87, 88] as well as in cell motility [89, 90], but the microscopic mechanisms by which an actomyosin network contractility emerges are still poorly understood. As reviewed by Murrell et al. [91], a number of theories have been proposed for how non-sarcomeric actomyosin networks generate contractile motion. In particular, the clustering of actin filament pointed ends, filament buckling under compressive loads, and active filament turnover via actin depolymerization and other biochemical regulators could play important roles in promoting actin filament orientations necessary to favor the contractile force generation of myosin II filaments, as opposed to an extensile force that could be produced without these mechanisms of geometric symmetry breaking [91]. Other related factors could also play large roles in this symmetry breaking for a randomly oriented network, including the overall deformability of myosin II and actin filaments [92], as well as the favoring of contractile energy configurations of myosin II mini-filaments [23].

Cross-linking proteins are also essential for actomyosin contractility. By enhancing the length scale of transmitted contractile forces, cross-linking proteins can promote large-scale contraction at lower concentrations, but can also inhibit it at higher concentrations *in vitro* [93]. Cross-linking proteins might also serve to influence the geometry of the actomyosin network by promoting actin filament bundle formation in tandem with myosin II [94], as well as by promoting larger scale actomyosin structures necessary for macroscopic contractile behavior [95–97]. Prior computational works have investigated the effects of cross-linker concentration on various aspects of actomyosin network dynamics [24–26, 28, 36, 97], but these models did not include the distinct chemical and molecular transport processes that occur in an actomyosin network, including diffusion, actin filament assembly and turnover, and spatially-localized myosin and cross-linker binding and unbinding events, which may contribute to the self-organization, subsequent reorganization and the emergence of contractility in the actomyosin-cross-linker network *in vitro*. With the MEDYAN framework, an actomyosin network can be simulated while treating chemical and mechanical properties, as well as molecular transport, with equal detail and realism, thus producing a balanced model of the dynamic time evolution of this complex network.

In this example application of the MEDYAN model, we investigated the effects of NMIIA and *α*-actinin concentration on the morphological and contractile properties of both a smaller 1 × 1 × 1 *μm* ^3^ and larger 3 × 3 × 3 *μm* ^3^ sized cubic actomyosin network. Simulations were performed for a number of actomyosin systems with varying molar concentration ratios of both NMIIA and *α*-actinin relative to a fixed overall actin concentration, denoted as *R*_m:a_ and *R*_*α*:a_, respectively. We observed the distinct morphological changes in the network that come with varying these concentrations. We also discuss below our quantitative and qualitative observations about overall network contractility under various conditions and modeling assumptions, as well as actin filament bundling and phase transitions into these bundles.

The simulated actomyosin systems are comprised of actin filaments that are coarse-grained into cylindrical segments as outlined in the Mechanical model section. Using harmonic potentials to represent the stretching and bending response under stress, F-actin filaments can be displaced when acted on by an external force due to NMIIA or an external boundary. Filamentous force constants were chosen based on the known persistence length and elastic modulus of actin filaments [98, 99]. Chemically, the filaments can polymerize and depolymerize with diffusing actin monomers at either end at specified rates determined experimentally [57]. These events increase or decrease the filament length by 2.7 *nm*, correspondingly [100]. To include the mechanochemistry of actin polymerization, we describe the polymerization rate change of a filament tip under external load with the Brownian Ratchet model as in previous works [101], as elaborated in Section C of S3 Text. Binding events that can occur on the actin filament will be described in more detail below, and we assume there is one binding site per 27 *nm* of actin filament, which can be dually occupied by *α*-actinin and NMIIA mini-filaments. We will discuss in a later part of this section the implications of this modeling decision and the consequences of introducing a mutual site exclusion of the cross-linkers and molecular motors.

The simulated actomyosin systems contain diffusing NMIIA mini-filaments that are assumed to contain 10–30 individual myosin heads [78] with each having an individual step size of *d*_*step*_ = 6 *nm* [102]. While we do not account for the explicit binding and walking of separate subunits to actin filaments in the ensemble, we have adopted the results of the Parallel Cluster Model of Erdmann et al. [103] to describe, in a coarse-grained fashion, the binding, unbinding, and walking of small ensembles of NMIIA motors in a similar manner to [36]. This model is fully mechanochemical and contains relationships for all reaction events in terms of the mechanical force acting on the mini-filament. See Section A of S3 Text for a detailed derivation of adopting the results of the Parallel Cluster Model to our coarse-grained description. When a NMIIA mini-filament stochastically binds to neighboring actin filaments within a distance of 200 ± 25 *nm*, which was chosen based on the known length of NMII mini-filaments [104], it creates a harmonic potential as outlined in the Mechanical model section, where the force constant for this potential has been chosen based on single molecule pulling experiments [102]. The walking of these head ensembles along filaments then can produce mechanical stress due to this potential, which actively remodels the actin network. The systems also contain diffusing *α*-actinin which can stochastically bind, and unbind to neighboring actin filaments within 35 ± 5 *nm*, which was chosen based on the known length of *α*-actinin [105]. When bound, the cross-linker creates a harmonic potential as described in the Mechanical model section with force constant found in single molecule pulling experiments [106]. To model the mechanochemical effects of pulling forces on *α*-actinin, a simple slip bond form was used; see Section B of S3 Text for more description on this mechanochemical model.

For each concentration configuration presented in the following sections, omitting the larger systems which will be presented later, 2000 *s* of simulation were run in a 1 × 1 × 1 *μm* ^3^ cubic spatial boudary with a hard-wall potential as outlined in the Mechanical model section at an actin concentration of 20 *μM*. The simulations all initially nucleate 50 short actin filaments at random positions and orientations, then grow them for 10 *s* in the presence of diffusing G-actin and *α*-actinin. Subsequently, diffusing NMIIA mini-filaments are added to the system. While the average actin filament length in these simulations is 0.8 *μm*, which may be shorter than the average actin filament length observed in vivo [107], this filament length is of relevance to the remodeling of lamellipodial and lamellar actin fragments by myosin II and cross-linking proteins. These fragments have been observed to be of lengths ranging from 0.5 to 2 *μm* in the lamellipodium under various conditions [108]. Sixteen trajectories were run for each actomyosin configuration, and all shown results are averaged over those trajectories.

### Network contractility controlled by R_m:a_ and R_α:a_

**Fig 5:**
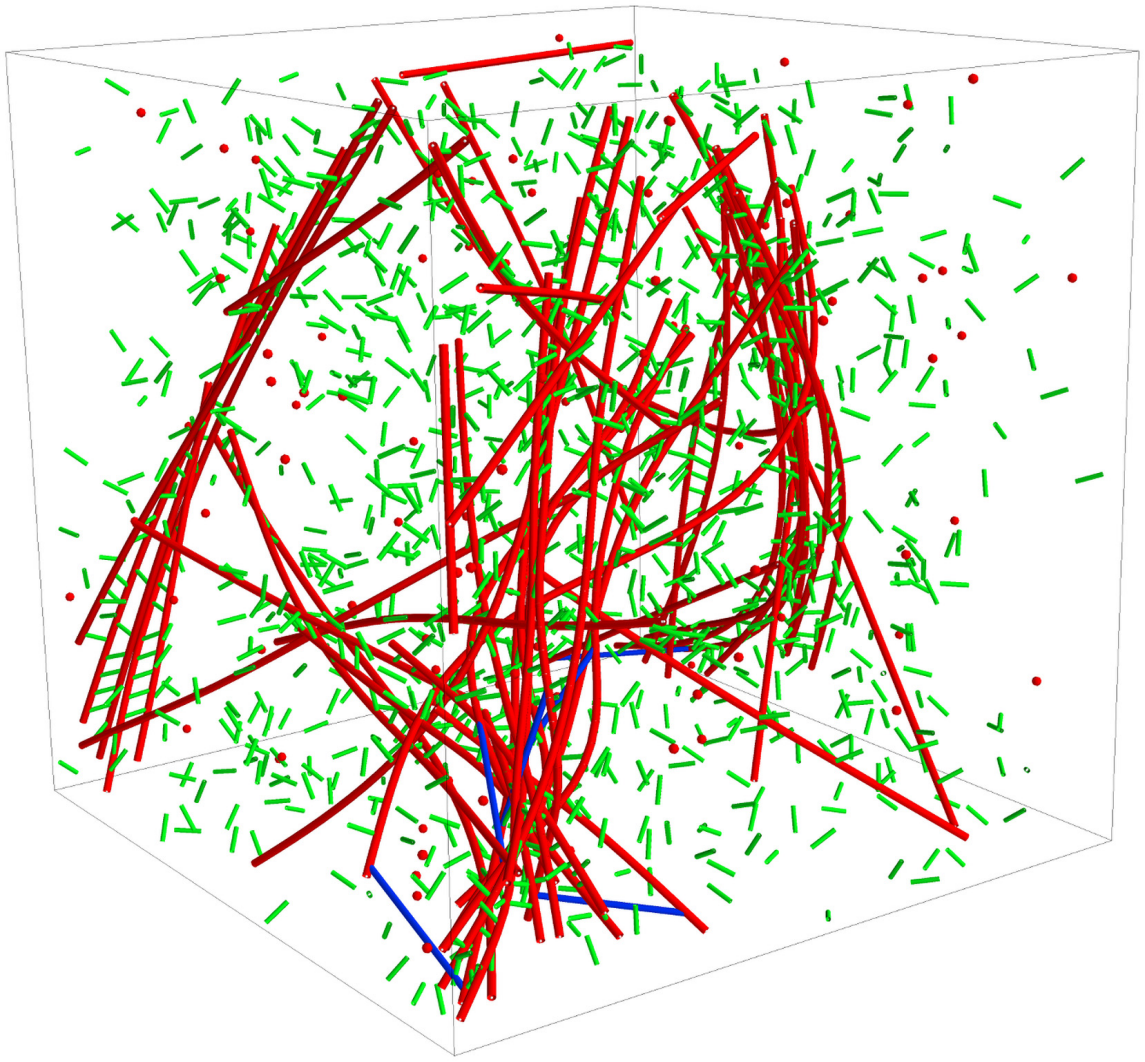
A single trajectory snapshot of a 1 × 1 × 1 *μ*m^3^ actomyosin system simulation at R_α:a_ = 0.1 and R_m:a_ = 0.01 after 2000 s of network evolution. Actin filaments are represented as red connected cylinders, *α*-actinin are represented as green cylinders, and NMIIA mini-filaments are represented as blue cylinders. The corresponding diffusing species are also shown in the same colors. The system is bounded by a cubic, hard-wall potential.

Fig 5 shows a single trajectory snapshot of an actomyosin system simulation containing actin filaments, NMIIA mini-filaments, *α*-actinin, and the constituent diffusing species within the simulation boundary. To define a quantitative measure of overall contractile behavior of the various actomyosin systems, we define an actomyosin network radius of gyration using all coarse-grained actin filament cylinder segments in the network:

$$R_{\text{g}}=\sqrt{\frac{1}{n}\sum_{i=0}^{n}{\left(r_{i}-r_{\text{GC}}\right)}^{2}},$$ (13)

where *r*_GC_ is the geometric center of the ensemble of cylinders, *r*_*i*_ is the position of the *i* ^*th*^ cylinder, and *n* denotes the number of cylinders in the network. This is a more useful measure for our system than other more macroscopic measurements, including contractile velocity and minimum enclosing spherical volume. This is due to the fact that the dynamics of most networks studied do not show an obvious volume contraction, but do reorganize rapidly into contractile structures. See S1 Fig for a set of visual examples describing the relationship between *R*_g_ and contractile network morphology.

**Fig 6:**
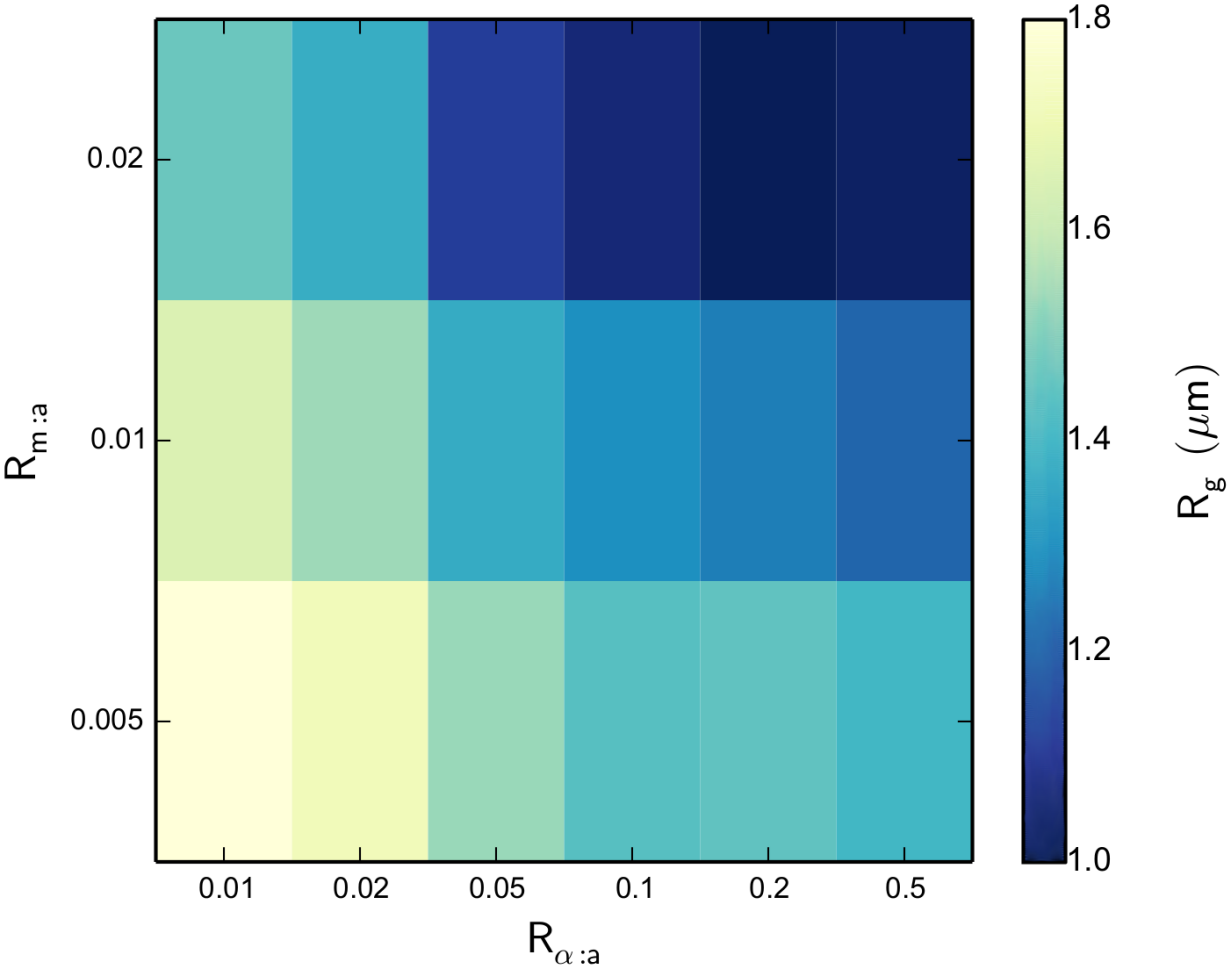
A heat map of actomyosin network R_g_ as a function of R_m:a_ and R_α:a_ after 2000 s of network evolution. As NMIIA and *α*-actinin concentrations are increased, a very apparent correlation in overall network contraction results.

**Fig 7:**
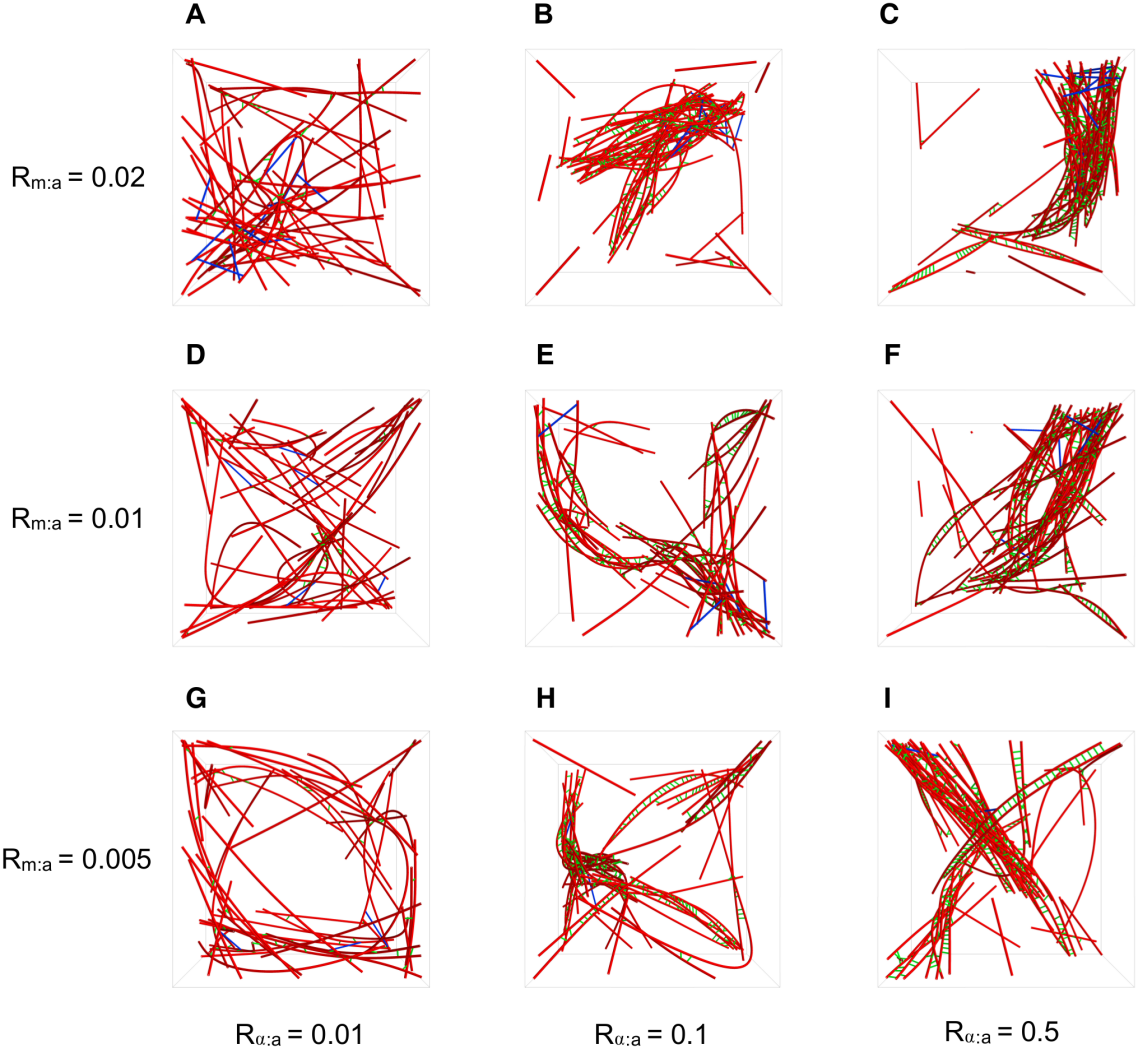
Single trajectory snapshots of the actomyosin systems with various concentrations of R_α:a_ and R_m:a_ after 2000 s of network evolution. These snapshots are shown without diffusing species for simplicity. For increasing *α*-actinin and NMIIA concentration, actin filament bundle formation is increasingly more apparent.

Fig 6 shows a heat map of actomyosin network *R*_g_ for the various systems after 2000 *s* of network evolution. We see some very obvious patterns, including a decreased *R*_g_ for increasing NMIIA concentration, which implies more contractile behavior with this increase. We also observe the same effect of decreasing *R*_g_ for increasing *α*-actinin concentration. These relationships make physical sense, as more motors can provide more contractile force and linkers aid this contraction by increasing the transmitted force length scale. Fig 7A–7I show the network morphology for various values of *R*_*α*:a_ as *R*_m:a_ is also varied. We observe that for the lowest value of *R*_*α*:a_ = 0.01, there is very little reorganization and contractile structure formation from the original randomly oriented network. But, with *R*_*α*:a_ values of 0.1 and 0.5, there is very apparent actin filament bundle formation. Increases in *R*_m:a_ throughout the systems tends to slightly increase the network’s ability to contract into more tightly packed structures, as was also indicated by the values of *R*_g_ in Fig 6.

### Switch-like transition to contraction above threshold R_α:a_

**Fig 8:**
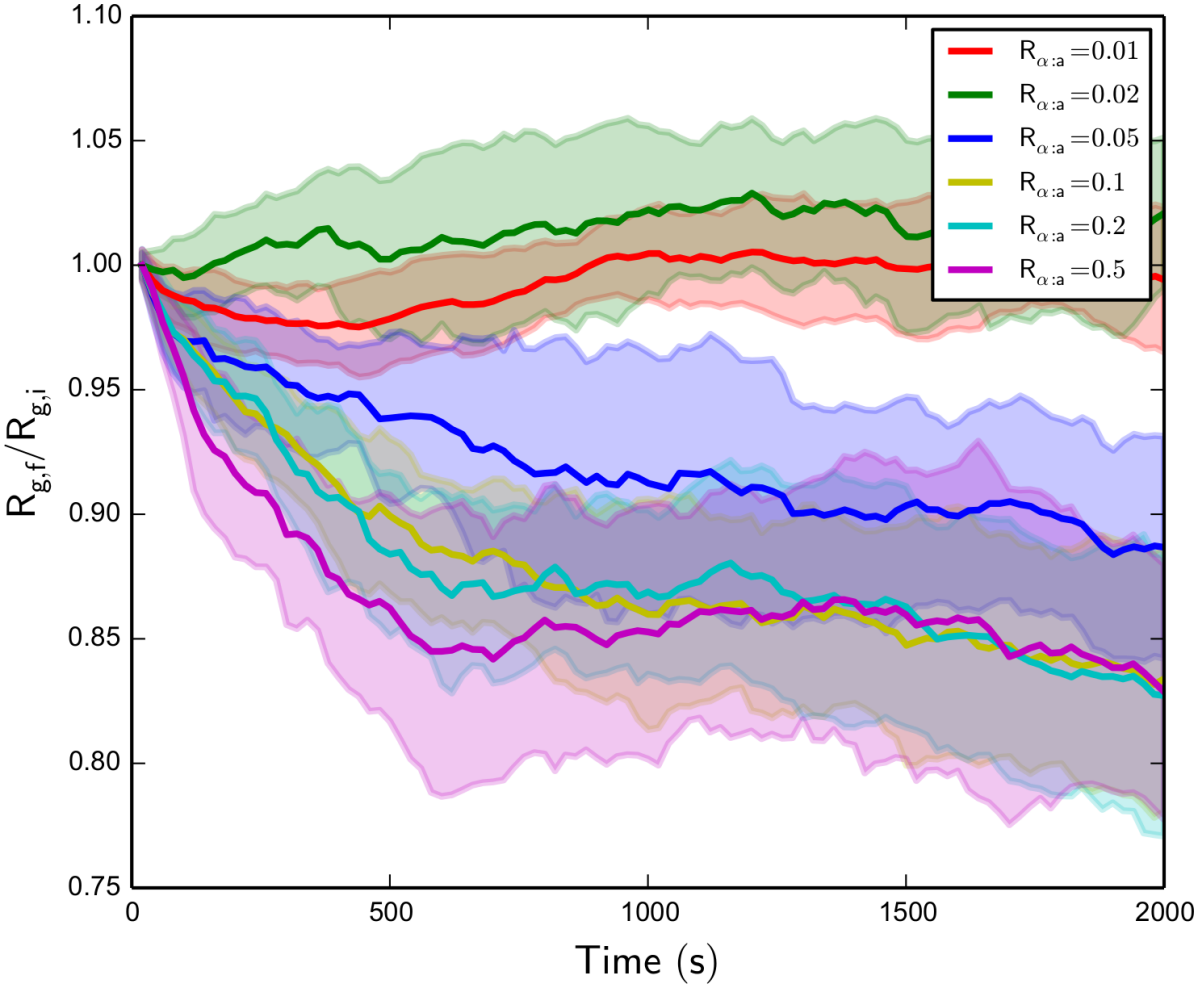
Actomyosin network R_g,f_/R_g,i_ over time for various R_α:a_ with fixed R_m:a_ = 0.01. We see that above a threshold *α*-actinin concentration, contraction is observed, and the time of bundle formation for these contractile structure formations decreases with increasing *α*-actinin concentration. Standard deviations of the *R*_g,f_/*R*_g,i_ values over all trajectories are shaded.

Since the final network *R*_g_ does depend on the initial configuration of the randomly oriented network, especially for non-contracted networks, it is useful to look at the ratio of final *R*_g_ after 2000 *s* to initial configuration *R*_g_ for the various systems, denoted as *R*_g,f_/*R*_g,i_. Fig 8 shows this value for a range of systems, holding *R*_m:a_ fixed, over the 2000 *s* of simulation time. We see that there is a clear divergence in time evolution for the lowest *R*_*α*:a_ values compared to the other higher values. This may imply, coupled with morphology observations of the various trajectories as in Fig 7A–7I, that there is not a continuous distribution of achievable contractile structures accessible with a given *R*_*α*:a_ and *R*_m:a_ as implied by Fig 6, but only at a certain minimum *α*-actinin concentration, actin filament bundle formation is possible. This also seems likely due to the fact that the systems with *R*_*α*:a_ values of 0.1, 0.2, and 0.5 converge to a similar *R*_g_ value after the entire simulation. Comparing this observation to other systems with different *R*_m:a_, we see that as motor concentration is increased, the minimum *α*-actinin concentration for actin filament bundle formation decreases, possibly due to the increased contractile strength of adding more NMIIA mini-filaments to the system.

From these observations, we deduce that in these systems, actomyosin system cross-linker concentration is a switch-like mechanism that controls a transition between disordered and bundled networks, with system motor concentration widening this contractile structure formation regime, thus decreasing the minimum cross-linker concentration needed for bundle formation. This result is in agreement with the predictions of cross-linker percolation theory in larger scale actomyosin networks [28, 95]. The formation of bundles agrees with the zippering mechanism of actin filament alignment and myosin II aggregation as proposed by Verkhovsky et al. [78, 94]. But, our results show that their is a more active role of cross-linkers in the process than was previously suggested: if cross-linker concentration is decreased below a certain threshold value, this cross-linker binding propensity decrease hinders actin filaments from being adequately zippered onto the existing bundle before another contractile myosin II can pull in another direction, or filament turnover drifts it away from that position, disallowing global bundle formation.

### Actin filament polarity alignment by NMIIA is regulated by filament turnover

Other interesting morphological properties of the contractile actomyosin networks were observed. S1 Video shows a single trajectory for a smaller actomyosin system with *R*_m:a_ = 0.01 and *R*_*α*:a_ = 0.1. When we color the plus and minus ends of these filaments with black and white beads in the same trajectory, respectively (see S2 Video), we see that the actin filaments within the bundle are globally aligned in polarity. This is an interesting result, especially since the actin filament network started with random orientation within the uniform spatial boundary condition, and has not been predicted by previous models of bundle formation by way of zippering [78, 94], which describe the resulting apolar alignment of actin filaments, but not polarity alignment. The physical origins of this global polarity alignment by NMIIA mini-filaments is unclear from the trajectory videos, but has been observed and analyzed in two-dimensional motility assays [109–111], as well as been predicted and modeled in one-dimensional actomyosin bundles undergoing polarity sorting [112, 113]. Constant turnover in the plus end direction of actin filaments most likely causes anti-parallel orientations to be unstable, thus in the long-time limit of our simulations, only parallel bundles survive. But, the observed global contraction implies that NMIIA mini-filaments are driving network dynamics to a globally aligned, contractile state. It is reasonable to assume that combination of these two factors attributes to the observed behavior.

To further quantify actin filament alignment in the simulated actomyosin networks, we define an orientational order parameter *S* of the system of actin filaments, which is the largest eigenvalue of the ordering tensor *Q* [114] which is constructed from

$$Q_{\alpha \beta }=\frac{3}{2}\left(\frac{1}{N}\sum_{i=0}^{N}u_{i\alpha }u_{i\beta }-\frac{1}{3}\delta_{\alpha \beta }\right),\;\text{where}\;\alpha ,\beta =x,y,z.$$ (14)

**Fig 9:**
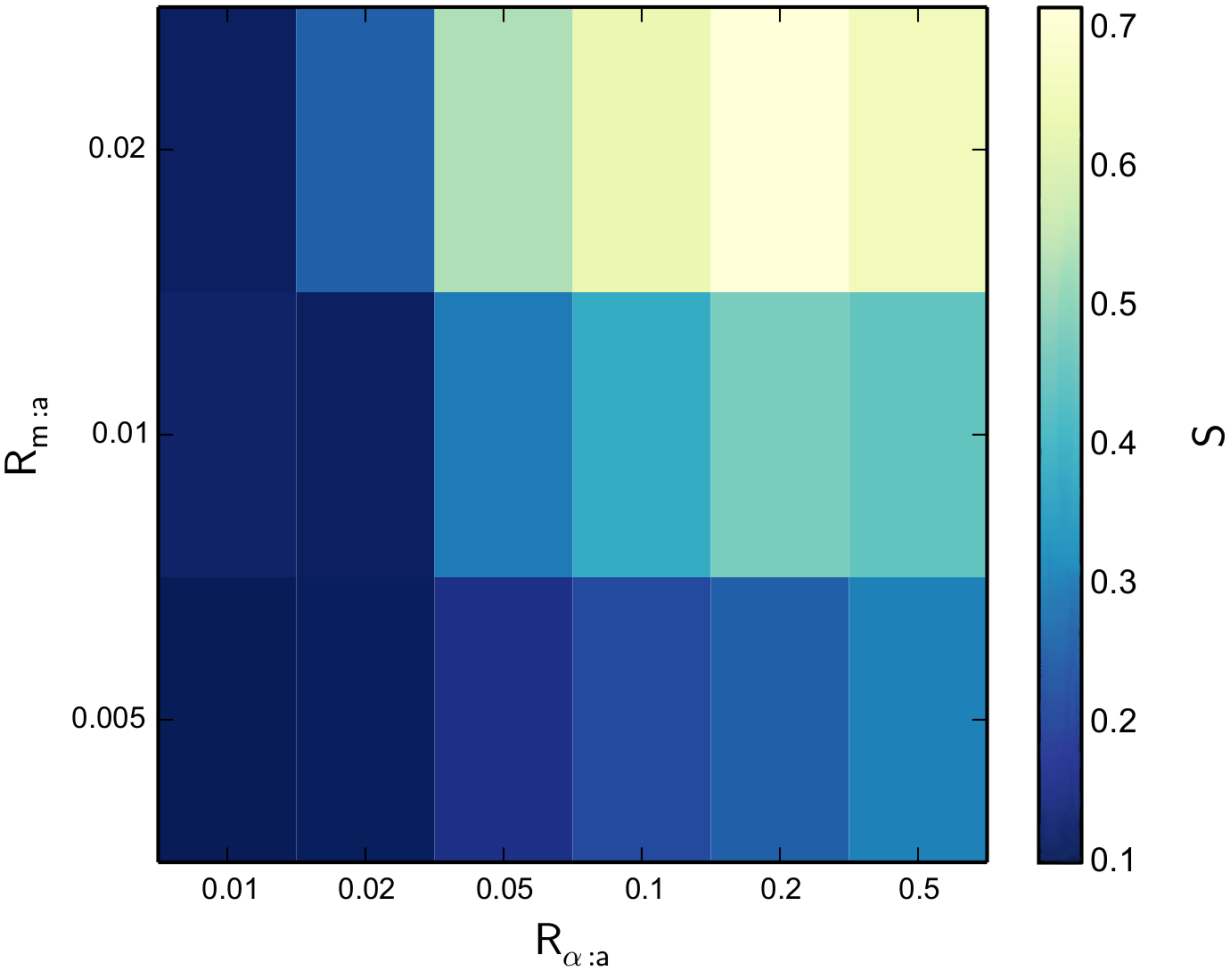
A heat map of actomyosin network S as a function of R_m:a_ and R_α:a_ after 2000 s of network evolution. As NMIIA and *α*-actinin concentrations are increased, a correlation in alignment results in a similar fashion to *R*_g_ in Fig 6.

The vector **u**_**i**_ represents the normalized direction of filament *i* over the *N* filaments in the system. When *S* is equal to zero, the filaments in the system are all randomly aligned, and when *S* equals 1, the filaments are all perfectly aligned, regardless of polarity. To numerically capture the alignment of bent actin filaments, we chose to use a direction vector from minus to plus end of the entire filament, as opposed to a calculation of *S* using cylindrical filament segments, which may give values corresponding to unaligned networks if an actin filament bundle is aligned but significantly bent in any direction. Fig 9 shows *S* for the various systems after 2000 *s* of network evolution, as *S* correlates directly with trends in *R*_g_ over the concentration ratios of *R*_m:a_ and *R*_*α*:a_, showing that all actomyosin systems produce alignment in tandem with contractile structure formation. We also confirmed qualitatively that in all actomyosin systems simulated, regardless of whether the systems eventually produced a single contractile bundle, all actin filament bundles formed consist of uniformly polar filaments, showing that all alignment observed is in fact polarity alignment.

**Fig 10:**
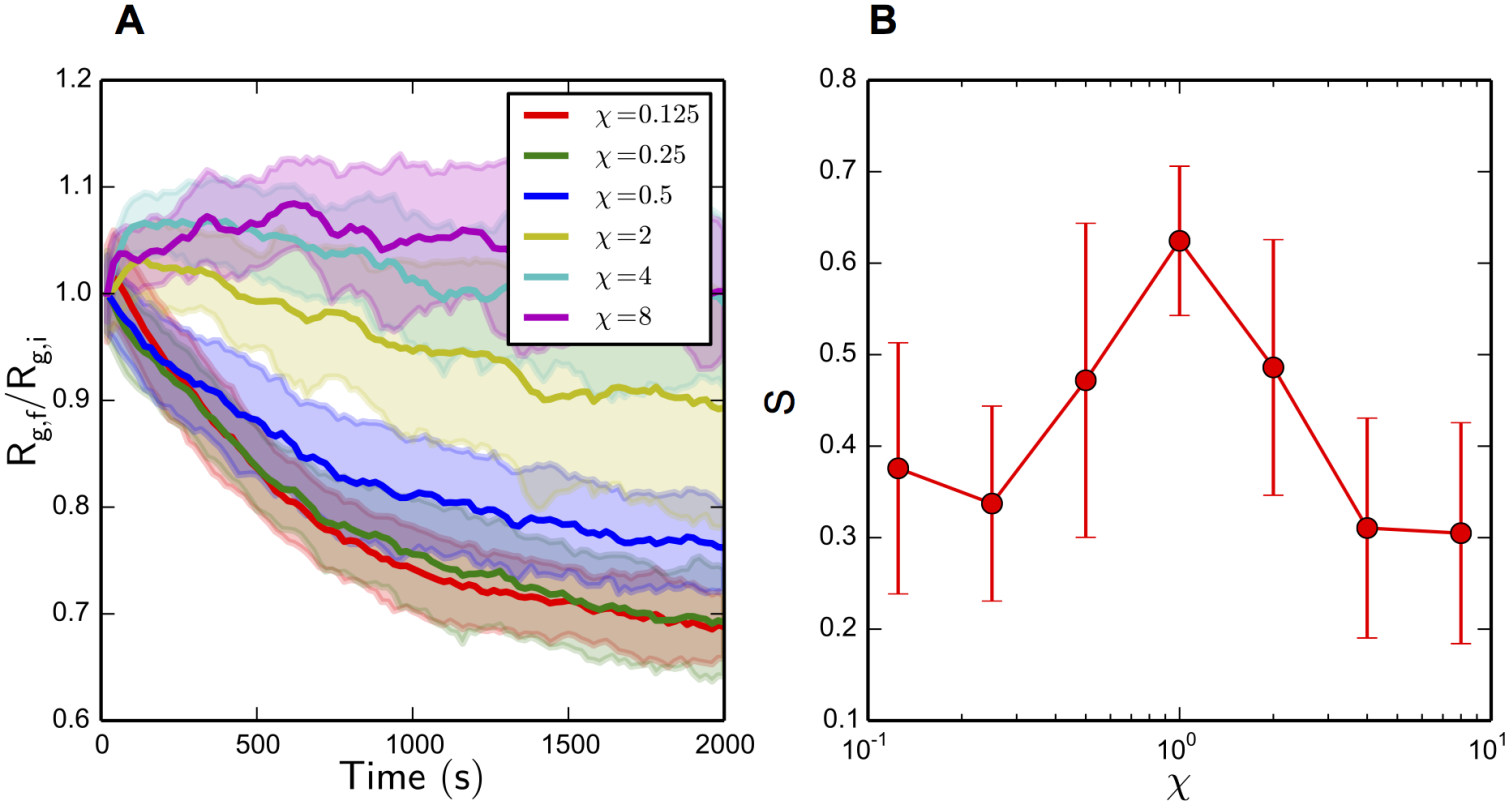
Actomyosin network R_g,f_ / R_g,i_ and S for various *χ*. (A) *R*_g,f_/*R*_g,i_ over the 2000 *s* network evolution for varying values of *χ*. Contractile behavior increases with decreasing *χ*. Standard deviations of the *R*_g,f_/*R*_g,i_ values over all trajectories are shaded. (B) *S* after 2000 *s* of network evolution for varying values of *χ*. Global alignment peaks around *χ* = 0.5 to 2, and decreases for values outside of this range. Error bars represent standard deviation of *S* values over all trajectories.

**Fig 11:**
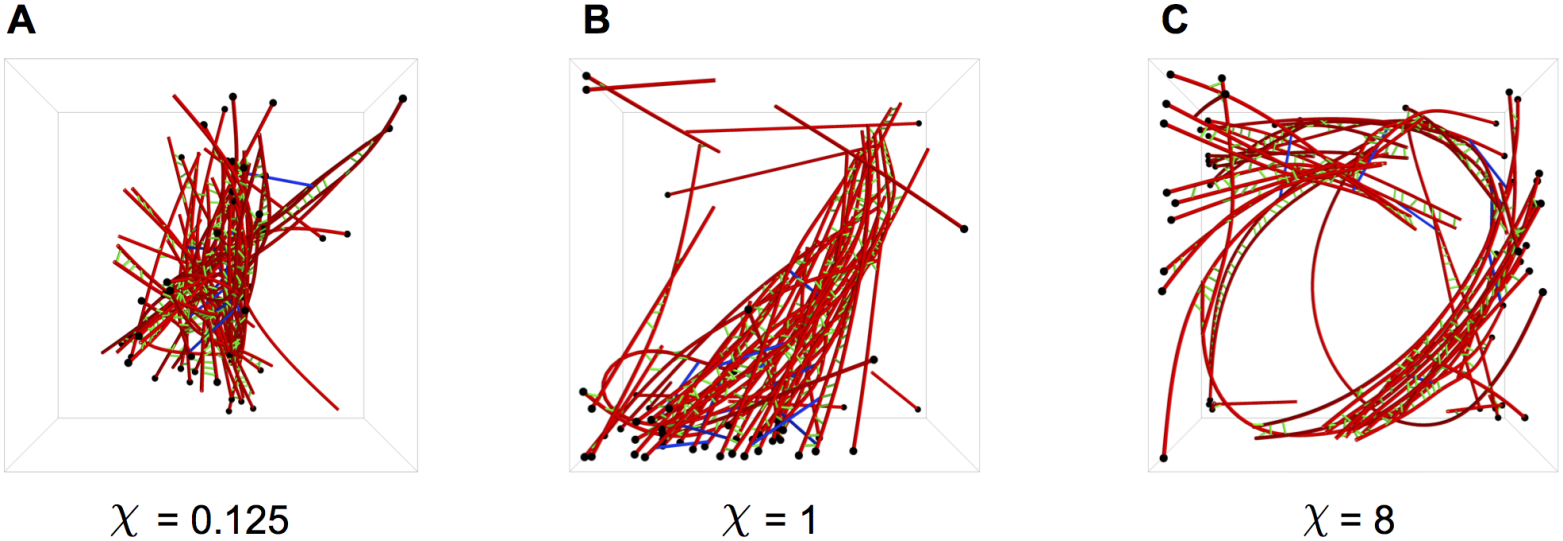
Single trajectory snapshots of the actomyosin systems, with various values of *χ* after 2000 s of network evolution. These trajectories are colored with black and white beads for the plus and minus ends of actin filaments, respectively. (A) Low *χ*, corresponding to a slow turnover rate, produces large global contractions in the absence of polarity alignment. (B) The original turnover rate (*χ* = 1) produces global contraction as well as polarity alignment. (C) High *χ* produces no global contraction, but distinct local polarity organization.

To probe the origins of this polarity alignment behavior which has been shown to be dependent on actin filament turnover in various systems [112, 113, 115], we vary the reaction constants used for polymerization and depolymerization of actin filaments *k*_actin,poly_ and *k*_actin,depoly_ at both the plus and minus ends of the filament by a constant factor *χ* while keeping *R*_m:a_ = 0.02 and *R*_*α*:a_ = 0.1 and holding all other parameter values constant. When this turnover factor *χ* is varied from 0.125 to 8, as shown in Figs 10 and 11, resulting in actin filament turnover rates of 0.07 to 4.4 monomers per *s*, distinct changes in network morphology result. At low *χ*, which corresponds to a very slow actin filament turnover rate, highly contracted networks are formed with little to no overall polarity alignment. In the case of high *χ*, no global contraction of the networks is observed, but local polarity alignment in small bundles is seen over the trajectories. Interestingly enough, the original parameters (*χ* = 1) which corresponded to physiological values of actin filament turnover, is the only parameter set to produce both global polarity alignment and contraction. Fig 11 shows the resulting network morphologies—low *χ* values corresponded to a dense, disordered clump with no polarity alignment, where high *χ* corresponded to local polarity alignment but overall disorder.

**Fig 12:**
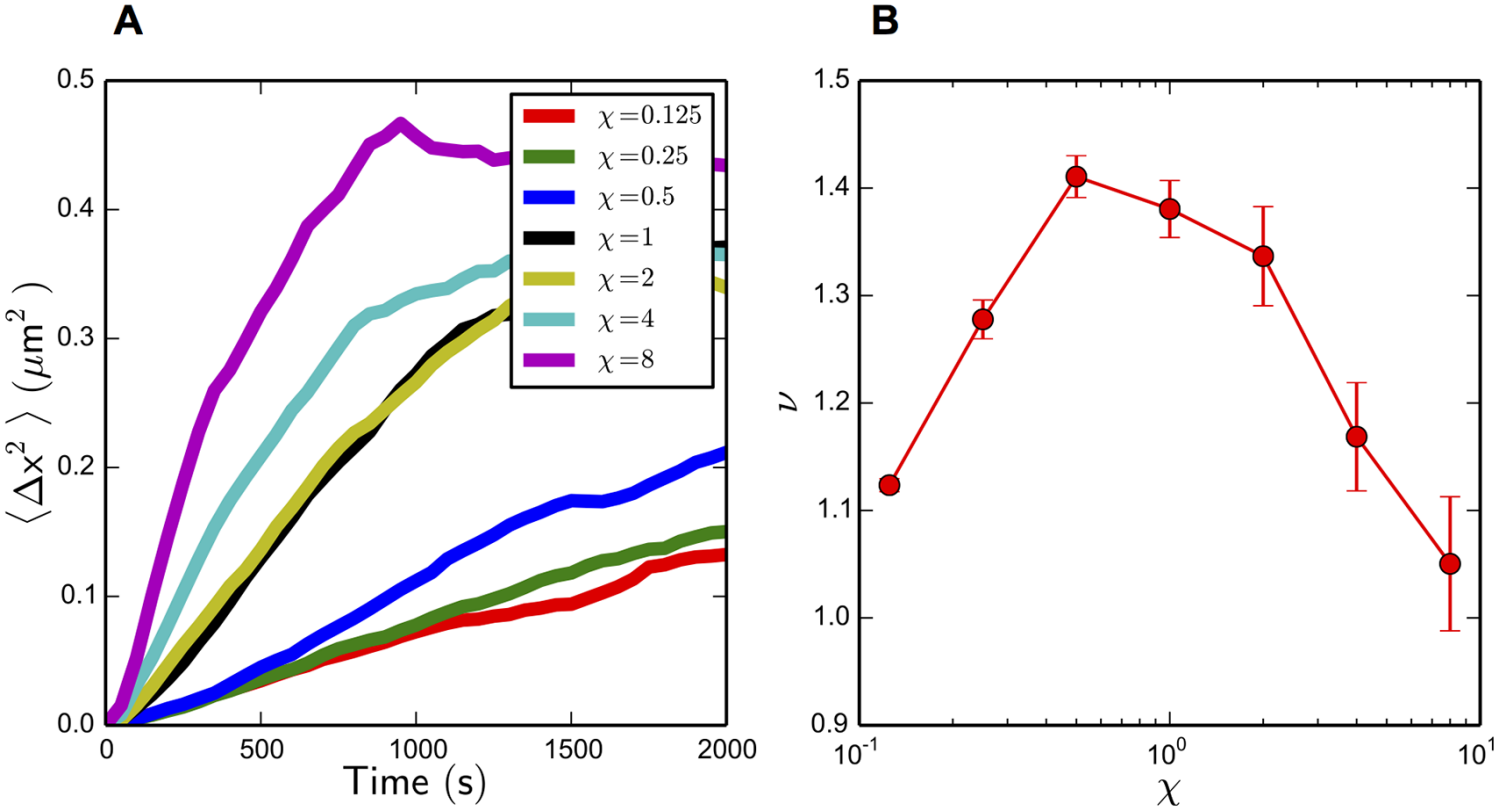
MSD analysis of actin filaments in simulation. (A) MSD over time for various values of *χ*. Error bars represent the standard error of the MSD, for each set of trajectories, are smaller than the data points. (B) Diffusion exponent *ν* acquired from a log-log linear fit of (A). Error bars represent the standard linear regression error in *ν*.

To investigate further the contraction and alignment dependencies found by varying the turnover factor *χ*, we look at the displacement of actin filaments over the time of the actomyosin system simulations, and compare different actin filament turnover rates to the resulting filament diffusivity. It is important to note that in this simulation context, actin filaments are not diffusing via Brownian motion in the simulation volume, but are actively moving via actin turnover and NMIIA mini-filament force generation, thus causing a relative displacement of the filament midpoint. Fig 12A shows the mean squared displacement (MSD) of actin filament geometric centers, denoted as 〈Δ*x* ^2^〉, with respect to simulation time over various *χ* values. To describe the motion of filaments under varying turnover rates, we linearly fit the first 1000 *s* of the MSD (choosing this cutoff due to kinetic arrest and sub-diffusion occurring after this time point, as shown in the sharp turns in MSD plotted against time for all *χ* in Fig 12A) on a log-log scale to obtain diffusion exponents, following the relation of general, anomalous diffusion:

$$\langle \Delta x^{2}\rangle ∼t^{\nu }.$$ (15)

The exponents *ν* corresponding to each turnover factor *χ* are shown in Fig 12B. All systems exhibited super-diffusion (*ν* > 1) in the 1000 *s* interval, which is physically reasonable due to the active nature of the many constituents. Surprisingly, the variation of *χ* resulted in a biphasic distribution with a maximum *ν* value centered around *χ* = 1, displaying the same *χ* dependence as the *S* distribution shown in Fig 10B. This relationship does make some intuitive sense; in a randomly oriented filament network, a higher-order filament diffusion relationship in any direction would cause anti-parallel filament bundle orientations to be less stable, thus producing a higher fraction of parallel bundles. But, it is counter-intuitive how the turnover factor *χ* affects the overall diffusive behavior in a biphasic manner. A physical explanation for the upper regime may be that when *χ* > 1, actin filament turnover can out-run displacements via NMIIA mini-filament walking, thus not allowing NMIIA remodeling at all, and producing locally aligned but globally disordered actin networks. But, as *χ* is increased while remaining under the threshold *χ* = 1, there is not a clear physical explanation for increased super-diffusive behavior and polarity alignment; a few studies have suggested actin filament turnover in the same direction of myosin II movement allows myosin II to walk farther on actin filaments, producing more contractile force [26, 116], but this is highly unlikely in our simulations due to the very short binding lifetime of NMIIA mini-filaments compared to actin filament turnover(about a 5 *s* unloaded NMIIA mini-filament attachment time compared to an average 0.5 monomers per *s* turnover rate), and furthermore, does not explain polarity alignment behavior. In fact, the actomyosin systems with the lowest *χ* values contracted more than ones with higher *χ*, as shown in Fig 10A. Nevertheless, our results suggest that filament movement in these systems, and thus polarity alignment, is a cooperative effect depending on the synergy of actin filament turnover and NMIIA mini-filament walking.

### Myosin II accumulates in areas of high network stress

We also notice the mechanochemical phenomena of NMIIA mini-filament force-dependent accumulation in both the smaller and larger system simulations where contractile behavior was observed. As shown in a smaller actomyosin system in S3 Video, after a stable actin filament bundle is formed, NMIIA mini-filaments accumulate at the base of the bundle where it is pushing forward into the boundary via actin filament turnover. This phenomena of the force-dependent accumulation of various cytoskeletal proteins was observed experimentally for cellular aspiration and further quantified by Luo et al. [84], and is responsible for many aspects of cellular mechanosensing. In particular, NMIIA has been shown to accumulate in areas of cytoskeletal stress, helping to maintain cytoskeletal integrity. In this case, the force-dependent accumulation of the NMIIA mini-filaments, being high-affinity cross-linkers when under stress, allows the bundle to maintain its structure when the pushing of the constituent actin filaments into the boundary may have disassembled the bundle. This spontaneous concentration gradient of NMIIA formed is a direct result of the coupling of diffusion of NMIIA mini-filaments and their force-dependent attachment affinity, which produces non-uniform compartmentalization within the simulation boundary.

### Larger system simulations exhibit polarity alignment and sorting

**Fig 13:**
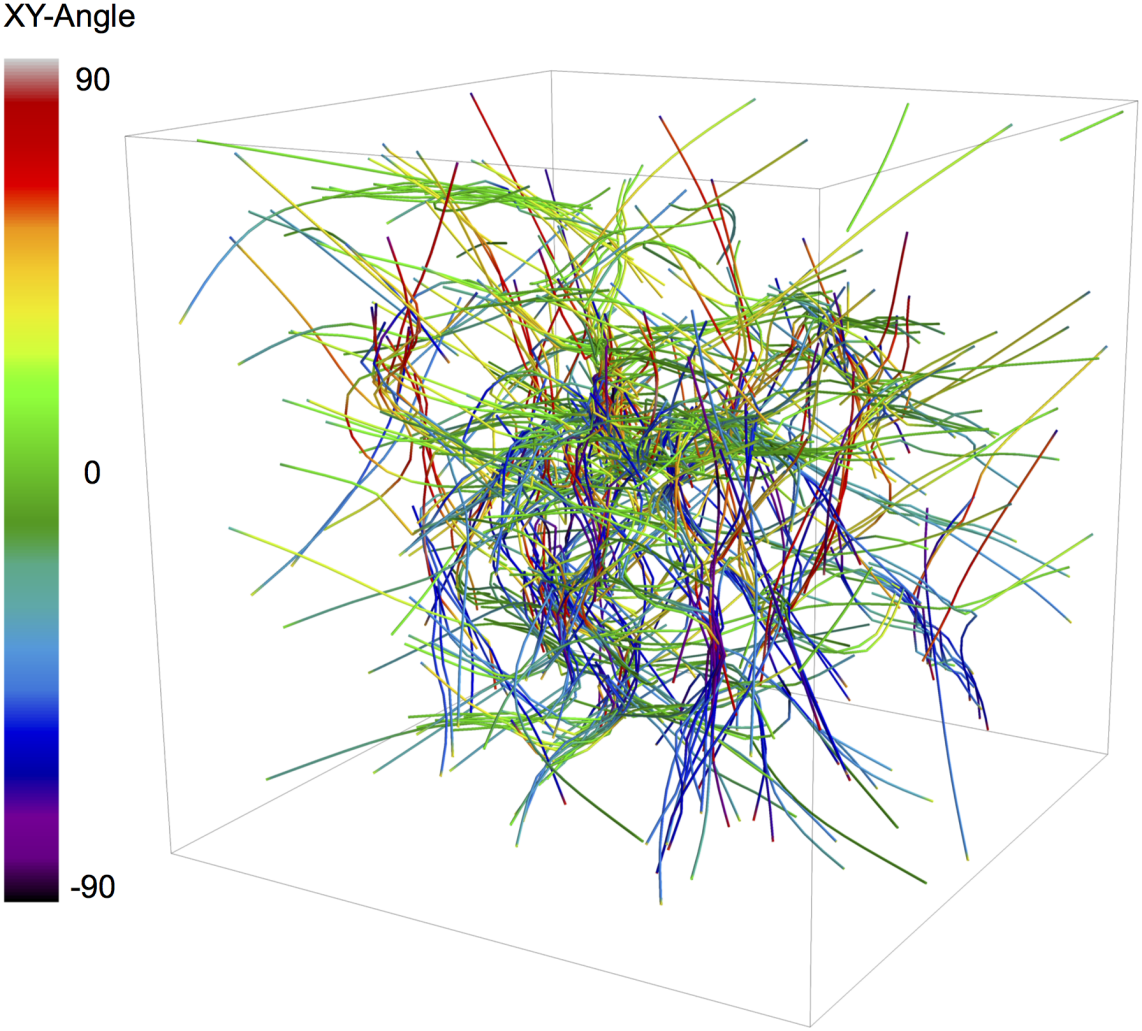
A single trajectory snapshot of a 3 × 3 × 3 *μ*m^3^ actomyosin system simulation at R_α:a_ = 0.1 and R_m:a_ = 0.02 after 500 s of network evolution. Actin filament cylinders are colored by their angle with respect to the x-y plane.

To test whether a larger, biologically relevant-sized system with longer actin filaments would undergo the same polarity sorting mechanisms as observed in the smaller 1 × 1 × 1 *μm* ^3^ systems, we ran another set of 16 trajectories, for 500 *s*, of a 3 × 3 × 3 *μm* ^3^ sized actomyosin network with an overall actin concentration of 12 *μM* and concentration ratios *R*_m:a_ = 0.02 and *R*_*α*:a_ = 0.1. 400 filaments were nucleated in the system, resulting in a mean actin filament length of 1.4 *μm* when reaching a steady-state actin concentration. A video of a single trajectory is shown in S4 Video. All trajectories did in fact undergo polarity alignment of sub-domains and overall sorting; Fig 13 shows a single trajectory snapshot of the actomyosin network with actin filament cylindrical segments colored by their directional angle with respect to the x-y plane. We see uniformly polar domains, with connections between those domains that span the entire simulation volume and appear to have similar polarity structure to sarcomeric bundle patterning observed *in vivo* [117]. Unfortunately, a detailed analysis of these larger systems is out of the scope of this paper and will be investigated in a future study.

### Binding site exclusivity limits contractile behavior

We note that at the highest values of *R*_*α*:a_, mainly at and above values of 0.5, which may be much higher than *α*-actinin concentrations seen at physiological conditions, network dynamics may be affected by our modeling assumption that *α*-actinin and NMIIA head ensembles can occupy the same binding site on an actin filament. At these high concentrations, one might assume that some kinetic arrest and motor jamming may occur, causing the NMIIA head ensembles to stall and not deform the network as freely as our results predict, possibly producing the biphasic regime of cross-linker concentration where actomyosin contractility is inhibited by further increasing it [93]. But, since these studies are on much larger systems that have actin networks which form multiple connected and bundled structures, it is unclear whether inhibitory cross-linker concentrations would appear at all on our smaller length scale.

In order to begin to study the effects of our binding occupancy assumption, we ran another set of simulations for all concentration configurations in a 1 × 1 × 1 *μm* ^3^ system that did not allow *α*-actinin and NMIIA head ensembles to occupy the same binding site on an actin filament cylinder. This resulted in an overall decrease in contraction for each concentration configuration, but produced slightly different trends in contractile behavior across values of *R*_*α*:a_ which seem to be slightly biphasic in nature; see S2 Fig for a heat map of network *R*_g_ after 2000 *s* of simulation, as well as *R*_g,f_/*R*_g,i_ over time for the various configurations. While this change in contractility trends across *R*_*α*:a_ did not seem to be statistically significant, we can hypothesize that the true competitive binding effects of cross-linkers and molecular motors lies somewhere between our two simulation extremes. A more detailed model of the steric exclusion of these molecules may give rise to a different contractile dependence on both *R*_*α*:a_ and *R*_m:a_.

## Discussion

Active matter is a growing field of study at the interface of chemistry, mechanics and non-linear physics. In order to model active networks with complete realism, a model must take into account not only chemical processes and the molecular transport that occurs, but also the mechanical response of the network as well as complex mechanochemical feedbacks that result. With the MEDYAN model, one is able to, in a flexible manner, simulate these entities with precision, while also explicitly accounting for their coupling. Having the powerful capability to simulate active networks with this amount of flexibility and detail in aspects of stochastic reaction-diffusion and coarse-grained polymer chain mechanics, this model could be used to provide additional insights on the mechanochemical dynamics of many active networks, including the cell cytoskeleton. To compare the MEDYAN model to other recent agent-based cytoskeletal modeling approaches, an extensive list of models in recent literature is given in S1 Table, with notes on the essential mechanochemical capabilities of each model as outlined in the Introduction.

Our public software implementation of MEDYAN (available at www.medyan.org) is also versatile enough such that other active networks, biological or artificial, could be simulated with a similar level of detail in comparison to its cytoskeletal applications, including self-organizing polymeric micelles [118], ParM polymerization mechanisms in bacterial mitosis [119], and many types of synthetic polymer gels. With these possibilities, the MEDYAN model is able to simulate a range of systems not previously achievable by other cytoskeletal models. Beyond the currently included chemical reaction set and mechanical force fields, the flexibility of the current software implementation also allows for the further development of the model to include new types of chemical and mechanical interactions as well as new classes of molecules, allowing for a completely customizable simulation framework.

As shown in the example application, simple actomyosin network simulations using the MEDYAN model can already capture the dynamics and shed light on the underlying mechanisms of actomyosin contraction and remodeling. Our results show that in a system consisting of actin filaments, myosin II mini-filaments, and cross-linkers, actin filament turnover and cross-linker concentration are both powerful tools to control actomyosin network reorganization and polarity alignment. These results have interesting implications for transverse arc assembly, which has been shown to be critically dependent on myosin II [78, 120, 121]: by way tightly regulating actin filament turnover as well as localized cross-linker concentration via biochemical regulators, a dynamic transition area between the lamellipodium and lamellum could form, where sharp changes in these parameters could result in dynamic network reorganization and bundle assembly in the lamellar region. The polarity alignment as well as network contraction via myosin II and actin filament turnover we have observed in our simulations suggests that a reorganization mechanism is occuring that is more complex than the previously proposed actomyosin zippering [78, 94], which predicts the apolar alignment of actin filaments but not polarity alignment. It is possible that the observed polarity alignment behavior in these simulations via myosin II and actin filament turnover could drive the sarcomeric polarity pattern formation seen in transverse arcs [122] when developing from an initially disordered, lamellipodia-like actin filament network. But, more studies on larger actomyosin networks with multiple bundled structures should be investigated in the future to test this polarity alignment and contraction mechanism.

While we observed contractile behavior in these systems as well as its dependence on cross-linker concentration, the exact contractile symmetry breaking mechanisms invoked in bundle formation, as well as the exact cooperation of actin filament turnover and myosin II mini-filament walking that results in actin filament polarity alignment, being difficult problems to analyze due to the many dynamic components of our simulation, remain unclear and will be further investigated in a future study. However, we hypothesize that cross-linkers may have an active role beyond increasing force transmission in overall contractile behavior due to the observed dependencies, and could break contractile-extensile symmetry by freezing contractile configurations into place by binding actin filament segments when they approach each other. We also propose that actin filament turnover may be a mechanism which allows actin filaments to flip and align in polarity more easily in the actomyosin-cross-linker system. Overall, our results show that in contractile systems where relevant timescales of motor movement are comparable to the timescale of network turnover, i.e. cross-linker (un)binding and actin filament turnover, interesting critical behavior can result, as shown in recent experiments [95, 123]. Determining the exact relationships between these timescales of importance at the observed critical points, as well as the resulting dynamic behavior and network reorganization in these systems, will be an interesting endeavor for cytoskeletal researchers in the future.

While the exclusivity of binding sites on actin filaments seems to alter the trends of contractile dependence of cross-linker concentration, more systems, as well as possible improvements to our model, should be studied in the future to probe these exclusion effects on network dynamics. In particular, we plan to include, in an explicit manner, a more realistic competition of cross-linkers and myosin II to binding sites on actin filaments. Also, the excluded volume effects of both molecules will be developed such that they cannot pass through actin filaments while network dynamics occur. These developments will help to study the dynamics of these actomyosin networks in a more realistic manner, and will provide additional insights to the problem of contractility emergence and mechanisms. The effect on the accumulation and kinetic trapping of myosin II mini-filaments when this steric exclusion is added will then be investigated, as it has interesting implications for myosin II compartmentalization within the cytoskeleton.

We note that the imposed spatial boundary conditions could play a role in the actin filament polarity organization observed, and, in tandem with NMIIA mini-filaments, might be a contributing factor to the observed uniform bundle polarities in the actomyosin systems. Future works could examine the role of spatial boundary conditions on these organized structure formations, as there have been interesting *in vitro* investigations of the effect of boundaries on actomyosin network assembly as reviewed by Vignaud et al. [124]. In particular, the effect of pre-defined actin network microarchitectures on myosin II dynamics could be further investigated [125].

## Supporting Information

S1 Text Model details. (A) Chemical model details, which includes descriptions of newly added chemical reactions. (B) Mechanical effects of the various chemical reactions that can occur in a simulation. (C) Mechanical minimization details, including the choice of key minimization parameters. (D) A description of the publicly available software implementation.
(PDF)

S2 Text Determining key simulation parameters for the actomyosin systems. (A) Calculation of the Kuramoto length and diffusion rate based on properties of the reaction network. (B) Choosing the gradient minimization tolerance based on force constants in the actomyosin system. (C) Choosing the number of chemical reaction steps per mechanical equilibration using properties of the reaction network and the mechanical effects of those reactions.
(PDF)

S3 Text Mechanochemical models used in the actomyosin systems. (A) Non-muscle myosin IIA, adopted from the Parallel Cluster Model. (B) *α*-actinin, chosen from single molecule experiments. (C) Actin filaments, using the Brownian Ratchet model.
(PDF)

S4 Text Validation and benchmarking of the MEDYAN model. (A) Validation of the optimized NRM for stochastic reaction-diffusion, and benchmarking performance of the original Gillespie algorithm compared to the optimized NRM in typical cytoskeletal systems. (B) Validation of the cylindrical coarse-graining scheme, with benchmarking performance tests in cases of typical cytoskeletal systems. (C) A note on the computational time used for the various simulations shown in the Results section, and time comparisons to previous models.
(PDF)

S1 Table A comparison of MEDYAN to other agent-based cytoskeletal models. These models are described in terms of their mechanochemical representations of cytoskeletal dynamics as outlined in the Introduction section.
(PDF)

S2 Table All simulation parameters used in the actomyosin systems. (A) Reaction rates, taken from experimental kinetic data. (B) Mechanical parameters, chosen to mimic actomyosin mechanics. (C) Mechanochemical parameters, adopted from single molecule experiments and the Parallel Cluster Model. (D) Other general parameters and their description.
(PDF)

S1 Video Single trajectory of a 1 × 1 × 1 *μ*m^3^ actomyosin system with R_m:a_ = 0.01 and R_α:a_ = 0.1. Actin filament bundle formation is observed. Bundle formation occurs mostly between *t* = 0*s* and 1500*s*, and is relatively stable afterward.
(MOV)

S2 Video Trajectory in S1 Video, colored with plus and minus ends of actin filaments as black and white beads, respectively. Uniform bundle polarity is observed. NMIIA mini-filaments and *α*-actinin cross linkers are not shown.
(MOV)

S3 Video Trajectory in S1 Video, with NMIIA mini-filaments colored by tension. NMIIA mini-filament force-dependent accumulation is observed after *t* = 1500, where the mini-filaments assemble at the base of the bundle where it is extending into the boundary. Actin filaments and *α*-actinin cross linkers are shaded in grey.
(MOV)

S4 Video Single trajectory of a 3 × 3 × 3 *μ*m^3^ actomyosin system with R_m:a_ = 0.02 and R_α:a_ = 0.1. The system is colored by actin filament cylinder angle with respect to the x-y plane. Cross-linkers and NMIIA mini-filaments are not shown. Bundling and polarity sorting of actin filaments is observed over the 500 *s* simulation.
(MOV)

S1 Fig Using R_g_ as a measure of actomyosin network contractility. (A) A *R*_g_ of 1.77 *μm*. (B) A *R*_g_ of 1.51 *μm*. (C) A *R*_g_ of 1.22 *μm*. Corresponding to a decrease of *R*_g_ from left to right, the actomyosin networks show more contractile structure formation. These snapshots are shown without monomeric actin, *α*-actinin, and NMIIA mini-filaments for simplicity.
(PNG)

S2 Fig Analysis of a 1 × 1 × 1 *μ*m^3^ actomyosin simulation set with binding site exclusivity. (A) A heat map of actomyosin network *R*_g_ as a function of *R*_m:a_ and *R*_*α*:a_ after 2000 *s* of network evolution. (B) Actomyosin network *R*_g,f_/*R*_g,i_ over time for various *R*_*α*:a_ with fixed *R*_m:a_ = 0.01. We see that in changing this modeling assumption, overall *R*_g_ is reduced and its dependence on *R*_*α*:a_ is altered.
(PNG)
