# Supplementary material for: MEDYAN: Mechanochemical Simulations of Contraction and Polarity Alignment in Actomyosin Networks
Source: PLoS Comput Biol. 2016 Apr 27;12(4):e1004877. doi: 10.1371/journal.pcbi.1004877 (PMC4847874; doi:10.1371/journal.pcbi.1004877)
Supplement: S1 Text — (A) Chemical model details, which includes descriptions of newly added chemical reactions. (B) Mechanical effects of the various chemical reactions that can occur in a simulation. (C) Mechanical minimization details, including the choice of key minimization parameters. (D) A description of the publicly available software implementation. (PDF) [file pcbi.1004877.s001.pdf]

Supporting Information for

MEDYAN: Mechanochemical Simulations of Contraction and Polarity Alignment in Actomyosin Networks

Konstantin Popov, James Komianos, and Garegin Papoian

Model details.

A) Chemical model details.

Simulation space is divided into discrete compartments, with the Kuramoto length chosen in a similar manner as recent work [1]. We do not consider mechanical interactions between diffusing chemical species, or their exact positions within the compartments. The compartment size is fixed throughout the simulation. The polymers in the system do not move or slip in the simulation space unless acted on by an external force, which is controlled by the potentials outlined in the Mechanical Model. All reactions are chosen and controlled by the Gillespie algorithm [2, 3]. Diffusion (and other forms of transport), polymerization, capping, and branching nucleation reactions occur as in previous work [1, 4–7]. The chemical reactions new to the MEDYAN model are outlined below:

- If we denote L and M as cross-linker and motor species, respectively, the binding and unbinding reactions can be described as follows:

$$P_1 + P_2 + L \leftrightarrow P_1LP_2, \text{and} \tag{1}$$

$$P_1 + P_2 + M \leftrightarrow P_1MP_2, \tag{2}$$

  where P_1 and P_2 are both polymers. Both equations 1 and 2 assume that the polymers are within the reaction range of this cross-linker or motor species.

- A motor can walk along either polymer it is bound to with the following form:

$$M_n \leftrightarrow M_{n+1}, \tag{3}$$

  where n denotes the binding site on the polymer. This implicitly assumes that the next binding site to move to on the polymer is not occupied. The rates of these reactions can be affected by external stresses, which is described in S3 Text.

- A non-branching filament nucleation reaction can occur in the bulk where two monomer species bind to form a new polymer. This reaction can occur with the following form:

$$X_1 + X_2 \rightarrow P, \tag{4}$$

where X_1 and X_2 are any diffusing species in the system. This creates a new polymer P with chemical composition $X_1 X_2$. Nucleated polymers are placed randomly in the simulation space corresponding to the compartment location of their reactant monomers.

- Polymers can also be severed implicitly with the following form:

$$P_0 \rightarrow P_1 + P_2, \tag{5}$$

where P_1 and P_2 denote the polymers resulting from a split of polymer P_0. This reaction keeps in tact the chemical composition of the original polymer.

- In the case of actin filaments, monomers can undergo an aging reaction corresponding to the steps of F-actin ATP hydrolysis. This reaction takes the form:

$$X_{1,\mathrm{f}} \rightarrow X_{2,\mathrm{f}}, \tag{6}$$

where $X_{1,\mathrm{f}}$ and $X_{2,\mathrm{f}}$ represent two distinct chemical species in the filament.

B) Mechanical effects of various chemical reactions.

Most chemical reactions have distinct mechanical effects on the network. We outline the chemical reactions and their mechanical effect below:

- Polymerization and depolymerization can occur at either end of a polymer. When a polymerization event occurs, the equilibrium length l_0 will increase by a monomer size, denoted by l_{mon}. When l_0 has reached its maximum size, which we will denote by l_{cyl}, this cylinder stops growing and a new cylinder with $l_0 = l_{\mathrm{mon}}$ is created. Similarly, when a depolymerization event occurs, l_0 will decrease by l_{mon}, and when $l_0 = l_{\mathrm{mon}}$, the cylinder will be removed.

- Motor and cross-linker binding can occur on neighboring polymers. When a binding reaction occurs, the respective interaction is created between the two binding cylinders as outlined in the Mechanical Model. The positions α and β are determined stochastically at binding. When unbinding, the interaction is removed from the two cylinders.

- Motor walking can occur on a polymer. When a walking reaction occurs, the positional parameter α (or β, depending on which side of the motor is walking) will increase by some discrete value. The motor may change cylinders due to a walking reaction, which would effectively reset the positional parameter and move the motor onto the next cylinder int the polymer chain. This movement will in turn generate a stress in the motor, and will eventually be equilibrated.

- Branching nucleation can occur on a polymer. When a branching nucleation reaction occurs, a new cylinder with $l_0 = l_{\text{mon}}$ is created at a branching site on the mother cylinder, which is determined stochastically. The respective interaction is created between the two cylinders as outlined in the Mechanical Model. This branched cylinder can now polymerize or depolymerize normally at its leading end. When branch unbinding occurs, this interaction is removed between the two cylinders, effectively freeing the daughter polymer.

C) Mechanical minimization details.

Mechanical equilibration is performed with any choice of force fields (which are outlined in the Mechanical Model) after a number of chemical reaction steps. Mechanical equilibration is performed using a conjugate gradient energy minimization algorithm. The system can be minimized to any force minimization tolerance $g_{\text{tolerance}}$. Depending on the size and nature of the active network being simulated, g_{tol} as well as the timestep per mechanical equilibration, denoted as t_{minimize}, can be chosen accordingly. For examples of choosing these parameters for a simulated active network, see S2 Text, which describes choosing a reasonable $g_{\text{tolerance}}$ and t_{minimize} for the example actomyosin systems presented in the paper.

D) Software implementation

The MEDYAN model has been implemented in a serial C++ code which uses efficient data structures and object-oriented programming paradigms to simulate active networks with the scheme described in earlier sections. Fig 1 shows the implemented software's general workflow. Upon reading user input regarding chemical species and reactions, mechanical interactions, and mechanochemical feedback relationships of constituent elements defined in the system, as well as various simulation parameters and algorithm specifications for these interactions in a number of system input files, the simulation system is constructed with all necessary data structures needed for the updating and management of key simulation components. By allowing a flexible creation of simulation elements, interactions, and algorithms, this software implementation of the MEDYAN model is able to perform simulations for a range of active matter systems with customizable components under various user-defined conditions. Additionally, this software has been constructed in highly compartmentalized fashion such that modifications and additions to the individual mechanical and chemical components, including interactions as well as algorithms, can be easily implemented.

After the system is initialized with the above components, the system controller object, which acts as the simulation driver, is responsible for evolving the system in time via chemical and mechanical interaction controllers. These child controllers, which also are initialized using user-defined parameters and are responsible for executing the specified simulation algorithms for stochastic reaction-diffusion and mechanical equilibration, then iteratively evolve the simulation system in time. The time evolution and subsequent deformations and growth of the simulated network, including the addition or deletion of components and other interactions that may affect the structure of the network, are then updated in the system by the main controller using an efficient callback system. In this way, chemical reactions, as well as mechanical interactions and deformations, can efficiently update system elements only when needed.

This software package, including source code for the serial C++ MEDYAN implementation, documentation on usage and compilation, and a trajectory visualization tool, is publicly available for general scientific use (www.medyan.org). It is encouraged that users create new patches to the

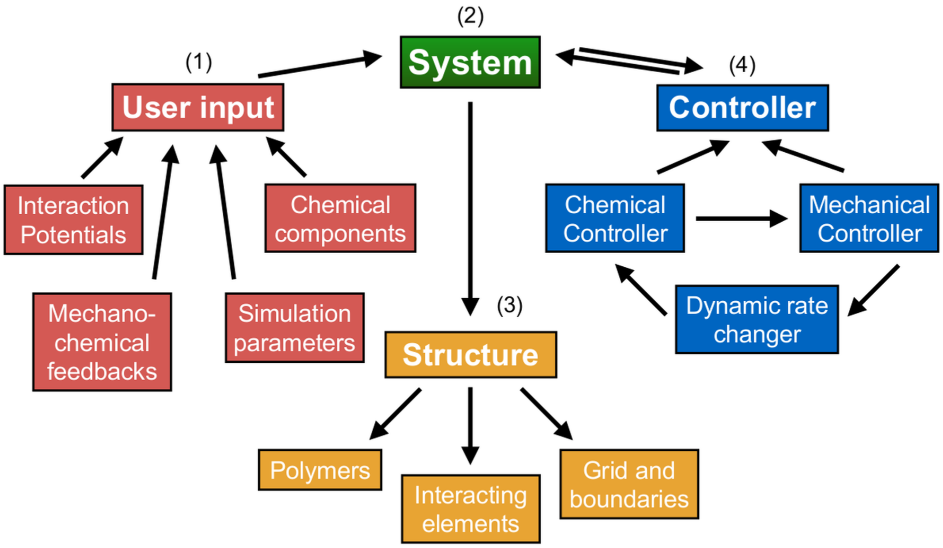

Figure 1: **A workflow diagram describing the MEDYAN software implementation.** (1) User input is given to create the (2) simulation system, which involves (3) setting up the necessary structures (4) and initializing the system controllers. During a simulation, (2-4) constantly communicate via an efficient callback system. We note that many less essential elements of the simulation workflow are omitted from this diagram.

existing code, tailoring it for many scientific purposes. In future work, this implementation will be made to run on parallel architectures, using some of the many available C++ parallelization tools, including the OpenMP and MPI libraries.

References

[1] Hu L, Papoian GA. Mechano-chemical feedbacks regulate actin mesh growth in lamellipodial protrusions. Biophys J. 2010;98(8):1375–1384.

[2] Gillesple DT. Exact stochastic simulation of coupled chemical reactions. J Phys Chem. 1977;93555(1):2340–2361.

[3] Gillespie DT. A general method for numerically simulating the stochastic time evolution of coupled chemical reactions. J Comput Phys. 1976;22(4):403–434.

[4] Lan Y, Papoian GA. The stochastic dynamics of filopodial growth. Biophys J. 2008;94(10):3839–3852.

[5] Hu L, Papoian GA. Molecular transport modulates the adaptive response of branched actin networks to an external force. J Phys Chem B. 2013;117(42):13388–13396.

[6] Zhuravlev PI, Lan Y, Minakova MS, Papoian GA. Theory of active transport in filopodia and stereocilia. Proc Natl Acad Sci. 2012;109(27):10849–10854.

[7] Zhuravlev PI, Papoian GA. Protein fluxes along the filopodium as a framework for understanding the growth-retraction dynamics: the interplay between diffusion and active transport. Cell Adh Migr. 2011;5(5):448–456.

[8] Bidone TC, Tang H, Vavylonis D. Dynamic Network Morphology and Tension Buildup in a 3D Model of Cytokinetic Ring Assembly. Biophys J. 2014;107(11):2618–2628.

[9] Tang H, Laporte D, Vavylonis D. Actin cable distribution and dynamics arising from cross-linking, motor pulling and filament turnover. Mol Biol Cell. 2014;25(19):3006–3016.

[10] Laporte D, Ojkic N, Vavylonis D, Wu JQ. Alpha-Actinin and fimbrin cooperate with myosin II to organize actomyosin bundles during contractile-ring assembly. Mol Biol Cell. 2012;23(16):3094–3110.

[11] Vavylonis D, Wu JQ, Hao S, O'Shaughnessy B, Pollard TD. Assembly mechanism of the contractile ring for cytokinesis by fission yeast. Science. 2008;319(2008):97–100.

[12] Nédélec F. Computer simulations reveal motor properties generating stable antiparallel microtubule interactions. J Cell Biol. 2002;158(6):1005–1015.

[13] Nédélec F, Foethke D. Collective Langevin dynamics of flexible cytoskeletal fibers. New J Phys. 2007;9(11):427.

[14] Kim T. Determinants of contractile forces generated in disorganized actomyosin bundles. Biomech Model Mechanobiol. 2014;14(2):345–355.

[15] Kim T, Gardel ML, Munro ED. Determinants of Fluidlike Behavior and Effective Viscosity in Cross-Linked Actin Networks. Biophys J. 2014;106(3):526–534.

[16] Jung W, Murrell MP, Kim T. F-actin cross-linking enhances the stability of force generation in disordered actomyosin networks. Comput Part Mech. 2015;2(4):317–327.

[17] Müller KW, Cyron CJ, Wall WA. Computational analysis of morphologies and phase transitions of cross-linked, semi-flexible polymer networks. Proc R Soc London A. 2015;471(2182):20150332.

[18] Cyron CJ, Müller KW, Schmoller KM, Bausch AR, Wall WA, Bruinsma RF. Equilibrium phase diagram of semi-flexible polymer networks with linkers. Europhys Lett. 2013;102(3):38003.

[19] Cyron CJ, Müller KW, Bausch AR, Wall Wa. Micromechanical simulations of biopolymer networks with finite elements. J Comput Phys. 2013;244:236–251.

[20] Cyron CJ, Wall WA. Finite-element approach to Brownian dynamics of polymers. Phys Rev E. 2009;80(6):1–12.

[21] Odell GM, Foe VE. An agent-based model contrasts opposite effects of dynamic and stable microtubules on cleavage furrow positioning. J Cell Biol. 2008;183(3):471–483.

[22] Rafelski SM, Alberts JB, Odell GM. An experimental and computational study of the effect of ActA polarity on the speed of Listeria monocytogenes actin-based motility. PLoS Comput Biol. 2009;5(7):e1000434.

[23] Alberts JB, Odell GM. In silico reconstitution of Listeria propulsion exhibits nano-saltation. PLoS Biol. 2004;2(12):e412.
