# Supplementary material for: MEDYAN: Mechanochemical Simulations of Contraction and Polarity Alignment in Actomyosin Networks
Source: PLoS Comput Biol. 2016 Apr 27;12(4):e1004877. doi: 10.1371/journal.pcbi.1004877 (PMC4847874; doi:10.1371/journal.pcbi.1004877)
Supplement: S2 Text — (A) Calculation of the Kuramoto length and diffusion rate based on properties of the reaction network. (B) Choosing the gradient minimization tolerance based on force constants in the actomyosin system. (C) Choosing the number of chemical reaction steps per mechanical equilibration using properties of the reaction network and the mechanical effects of those reactions. (PDF) [file pcbi.1004877.s002.pdf]

Supporting Information for

MEDYAN: Mechanochemical Simulations of Contraction and Polarity Alignment in Actomyosin Networks

Konstantin Popov, James Komianos, and Garegin Papoian

Determining key simulation parameters for the actomyosin systems.

A) Calculation of the Kuramoto length and diffusion rate.

The calculation of the Kuramoto length for the system of interest is as follows: assuming a diffusion constant D of 20 $\mu m^2 s^{-1}$ for G-actin as in previous work [1], the Kuramoto length l_k of the system is defined as

$$l_k = \sqrt{6D\tau}, \tag{1}$$

where $\tau$is the timescale of the fastest chemical reaction in the system. For this system, actin polymerization is the fastest reaction (see Table 1A in S1 Table for reaction rates used in the actomyosin systems), and with an initial concentration of $[A] = 20\ \mu M$, the timescale of this reaction is

$$\tau = \frac{1}{k_{\text{actin,poly,+}}[A]} = 0.003s, \tag{2}$$

giving a Kuramoto length of 0.64 μm. We will choose the compartment length to be 0.5 μm. It is noted that this is a highly conservative choice of compartment size, since eventually in simulation the actin concentration stabilizes around 0.2 μM. The rate of the diffusion reactions between compartments using this 0.5 μm length, with the diffusion rate as before, is 80 s^{-1}.

Unfortunately, there exists little data on the diffusion constants of α-actinin and NMIIA mini-filaments, especially in our reconstituted *in vitro* system. To calculate a diffusion rate between compartments for the these molecules, we will assume both of these molecules have a diffusion constant D that is a factor of 10 and 100 less than the diffusion constant of actin, respectively, due to their size and shape. Using the chosen compartment length, this will give a diffusion rate between compartments of 8 s^{-1} and 0.8 s^{-1}, respectively, for those molecules. It is noted that actin filaments of any length do not diffuse in our simulation.

B) Choosing the gradient minimization tolerance.

In order to determine an appropriate gradient tolerance $g_{\text{tolerance}}$ for mechanical equilibration, we have performed benchmarks in S4 Text to describe the performance and displacement error in polymer deformations under various $g_{\text{tolerance}}$. Based on these results, we chose a $g_{\text{tolerance}} = 1\ pN$, which produces sufficiently small displacement errors on the order of nanometers, which already is way beyond the desired resolution of this model.

C) Choosing the timestep between mechanical equilibration.

In order to determine an appropriate timestep between mechanical equilibrations, denoted as $t_{\text{minimization}}$, we can take into account the frequency of walking events of NMIIA mini-filaments along actin filaments, which will define a reasonable timestep between motor walking events, and thus the time allowed between mechanical minimizations in simulation, since this is the only molecule in the system producing large forces, and thus significant deformations in the network.

The zero-force walking rate between cylinders of an average-sized NMIIA mini-filament in our mechanochemical model is about 0.4 s^{-1}. With the given concentrations simulated, which correspond to between 2-8 NMIIA mini-filaments in the smaller actomyosin systems, it is reasonable to choose a $t_{\text{minimization}}$ of 0.1 s, which will correspond to 0.1-0.3 walking events per minimization, respectively. For the larger actomyosin systems, we will use the same timestep, as motor walking events will be distributed throughout the simulation volume and the chances of direct interactions of walking events between motors within $t_{\text{minimization}}$ is extremely unlikely.

References

[1] Hu L, Papoian GA. Mechano-chemical feedbacks regulate actin mesh growth in lamellipodial protrusions. Biophys J. 2010;98(8):1375–1384.
