# Supplementary material for: MEDYAN: Mechanochemical Simulations of Contraction and Polarity Alignment in Actomyosin Networks
Source: PLoS Comput Biol. 2016 Apr 27;12(4):e1004877. doi: 10.1371/journal.pcbi.1004877 (PMC4847874; doi:10.1371/journal.pcbi.1004877)
Supplement: S3 Text — (A) Non-muscle myosin IIA, adopted from the Parallel Cluster Model. (B) α-actinin, chosen from single molecule experiments. (C) Actin filaments, using the Brownian Ratchet model. (PDF) [file pcbi.1004877.s003.pdf]

Supporting Information for

MEDYAN: Mechanochemical Simulations of Contraction and Polarity Alignment in Actomyosin Networks

Konstantin Popov, James Komianos, and Garegin Papoian

Mechanochemical models used in the actomyosin systems.

It is noted before beginning this section that all values of reaction rates and mechanochemical parameters used in the mechanochemical models outlined below can be found in S1 Table.

A) Non-muscle myosin IIA.

Myosin II, in muscle and non-muscle isoforms, has been shown by many to have distinct mechanochemical properties that allow the molecule to respond to stresses in the cytoskeleton [1–4]. We outline a simple model proposed for myosin II mini-filament binding, unbinding, and walking in the case of non-muscle myosin IIA (NMIIA). Much of this work is an extension of the results for the Parallel Cluster Model of small, non-processive myosin motor ensembles introduced by Erdmann et al. [5], and is adopted to fit our coarse-grained description. We assume the following regarding mechanics for our implicit model of NMIIA mini-filaments:

- NMIIA mini-filaments are comprised of 10-30 implicit subunits [6], and the number of subunits is randomly chosen when a binding event occurs. Mechanical constants are recalculated accordingly based on the number of subunits selected. We will denote the number of subunits as N_{total}, since this value also represents the number of head groups (or heads) on each side of the bipolar filament.
- The forces that will occur in this network are not large enough for the NMIIA ensemble to exhibit slip bond behavior, so that the mechanochemical effect of increased pulling force will be a catch bond. The form of this relationship will be outlined below.
- The stiffness of a single NMIIA head has been determined experimentally by Vilfan et al. [7]. The stiffness of the entire mini-filament is then calculated based on the number of subunits.

We also assume the following regarding the single NMIIA cross-bridge cycle and associated chemistry:

- We assume a simplified cross-bridge cycle, as in work by Stam et al. [8] that has two states, bound and unbound:

$$\text{Bound state: } AM \cdot ADP$$

Unbound state: $M \cdot ADP \cdot Pi$

- The duty ratio ρ of NMIIA is low, and this duty ratio defines the mechanochemical effects of force on unbinding and walking, as will be elaborated on in the later section. We use the duty ratio and reaction rate values found by Kovacs et al. [9].
- The bound state of NMIIA is the only state that is mechanochemically affected. Isoforms of myosin II show a decreased dissociation rate of ADP when bound to actin with increased pulling force in the lower force regime [10]. The chosen form of this mechanochemical effect will be show below.

The binding rate of a NMIIA mini-filament to a pair of actin filaments can be defined as

$$k_{\text{fil,bind}} = \alpha \cdot k_{\text{NMIIA,bind}} \cdot N_{\text{total}} \tag{1}$$

since there are N_{total} NMIIA heads in the mini-filament that can bind to F-actin, and $k_{\text{NMIIA,bind}}$ is the binding rate of a single NMIIA head to an actin filament. α is a tunable parameter that can be chosen to fit NMIIA mini-filament binding kinetics; this will be elaborated on below.

Due to the molecule's catch bond behavior, the unbinding rate of a NMIIA mini-filament from a pair of actin filaments can be written as

$$k_{\text{fil,unbind}} = \frac{\beta \cdot k^0_{\text{NMIIA, unbind}}}{N_{\text{bound}}} \cdot \exp\left(\frac{-F_{\text{ext}}}{N_{\text{bound}} \cdot F_{\text{NMIIA,unbind}}}\right), \tag{2}$$

where F_{ext} is the total stretching force of the NMIIA mini-filament and $F_{\text{NMIIA,unbind}}$ is the characteristic unbinding force, determined by thermal energy and the NMIIA head unbinding distance. This relationship has been shown by [5]. β is another tunable parameter chosen to fit NMIIA mini-filament unbinding kinetics.

In our model, N_{bound}, the number of currently bound NMIIA heads to actin filaments, is implicit. The number of bound heads can be approximated to increase linearly with force in the regime we are considering [4, 5]. So, we can express N_{bound} as

$$N_{\text{bound}} = \rho \cdot N_{\text{total}} + \gamma \cdot F_{\text{ext}}, \tag{3}$$

where ρ is the duty ratio under no load, and γ, similarly to α and β, is a parameter chosen to fit NMIIA mini-filament unbinding kinetics.

As shown by Hill et al. [11] generally for motor ensembles, and nicely rearranged by [5], the walking rate of a NMIIA mini-filament under a constant external load can be written as

$$k_{\text{ens,walk}} = k^0_{\text{ens,walk}} \cdot \frac{F_{\text{stall}} - \frac{F_{\text{ext}}}{N_{\text{total}}}}{F_{\text{stall}} + \frac{F_{\text{ext}}}{N_{\text{total}}\zeta}}, \tag{4}$$

where F_{stall} is the stall force of a NMIIA head, and and F_{ext} is the pulling force on the NMIIA mini-filament in the direction opposite of walking movement, and ζ is a tunable parameter chosen to fit NMIIA mini-filament walking kinetics. Erdmann et al. showed that this parameter, as well as the stall force F_{stall} does not change for mini-filaments in the subunit range that we are considering [5].

In terms of our implicit model, $k^0_{\text{ens,walk}}$, which is the walking rate under no load, can be approximated, as by Erdmann et al., by using the number of unbound heads in the NMIIA ensemble, as well as the binding rate of a single NMIIA head [5]. So, we can express $k^0_{\text{ens,walk}}$ as

$$k^0_{\text{ens,walk}} = \frac{N_{\text{total}} - N^0_{\text{bound}}}{N^0_{\text{bound}}} \cdot k_{\text{NMIIA,bind}}, \tag{5}$$

where N^0_{bound} is the number of bound NMIIA heads under no load, which is simply ρN_{total}. It is noted that in our model, the walking rate must also be multiplied by a fraction of relative step size of a single motor head to the step size of the entire mini-filament in simulation, which is based on the number of binding sites per cylinder.

In choosing the mechanochemical parameters α, β, γ, and ζ to be 1.0, 0.2, $0.05/pN$, and 0.1, respectively, the mechanochemical model outlined above gives an unloaded attachment time of 5 s and an unloaded walking rate of 11 nm/s. Under stall conditions (i.e. $F_{\text{ext}} = \rho K_{\text{NMIIA,stretching}} d_{\text{step}}$), the model gives a loaded attachment time of 50 s and a loaded walking velocity of 3 nm/s.

B) α-actinin.

α-actinin is a cross-linking protein typically found in the cytoskeleton that has distinct mechanical and chemical properties. We outline a simple model for representing the mechanochemistry of this cross-linker, including binding and unbinding.

We assume the following regarding mechanics for our model of α-actinin cross linkers:

- The stiffness of α-actinin has been experimentally determined by Ferrer et al. [12].
- We ignore any unfolding domains and assume a constant length. Although there are known unfolding domains that may change network elasticity [13], these will not be included in our model. Forces in our network also will not be large enough to cause these unfolding events.

We also assume the following regarding α-actinin binding and unbinding chemistry:

- Binding occurs at a constant rate unaffected by force, and has been experimentally determined by Wachsstock et al. [14].
- Unbinding is mechanochemically affected, and we will assume that it is a simple slip bond. The form of this mechanochemical effect will be shown below.

The rate of unbinding will be affected by the pulling force on the cross-linker, and can be expressed as

$$k_{\alpha,\text{unbind}} = k^0_{\alpha,\text{unbind}} \cdot \exp\left(F_{\text{ext}}/F_{\alpha,\text{unbind}}\right), \tag{6}$$

where $k^0_{\alpha,\mathrm{unbind}}$ is the α-actinin unbinding rate under no external load, F_{ext} is the pulling force on the molecule, and $F_{\alpha,\mathrm{unbind}}$ is the characteristic unbinding force of α-actinin, determined by thermal energy and the α-actinin unbinding distance.

C) Actin filaments.

Actin filaments are dynamic species which can polymerize and depolymerize from either end. We outline the mechanochemical effect of filaments polymerizing under an external load, provided by a boundary. We assume the following regarding the mechanics of actin filaments:

- The stiffness and flexural rigidity has determined by experiments [15, 16].
- Both ends of the filament can experience an external load force from a boundary.

The polymerization rate of a filament tip under external load can be modeled by the Brownian Ratchet [17] with the following form:

$$k_{\mathrm{poly}} = k^0_{\mathrm{poly}} \cdot \exp\left(-F_{\mathrm{ext}}/F_{\mathrm{actin,poly}}\right), \tag{7}$$

where k^0_{poly} is the polymerization rate under no external load, F_{ext} is the external load force on the actin filament, and $F_{\mathrm{actin,poly}}$ is the characteristic polymerization force of an actin filament, determined by thermal energy and the size of an actin monomer.

References

[1] Nishizaka T, Seo R, Tadakuma H, Kinosita K, Ishiwata S. Characterization of single actomyosin rigor bonds: load dependence of lifetime and mechanical properties. Biophys J. 2000;79(2):962–974.

[2] Kovács M, Thirumurugan K, Knight PJ, Sellers JR. Load-dependent mechanism of nonmuscle myosin 2. Proc Natl Acad Sci. 2007;104:9994–9999.

[3] Luo T, Mohan K, Iglesias PA, Robinson DN. Molecular mechanisms of cellular mechanosensing. Nat Mater. 2013;12(11):1064–71.

[4] Piazzesi G, Reconditi M, Linari M, Lucii L, Bianco P, Brunello E, et al. Skeletal Muscle Performance Determined by Modulation of Number of Myosin Motors Rather Than Motor Force or Stroke Size. Cell. 2007;131:784–795.

[5] Erdmann T, Albert PJ, Schwarz US. Stochastic dynamics of small ensembles of non-processive molecular motors: The parallel cluster model. J Chem Phys. 2013;139(17):175104.

[6] Thoresen T, Lenz M, Gardel ML. Thick filament length and isoform composition determine self-organized contractile units in actomyosin bundles. Biophys J. 2013;104(3):655–665.

[7] Vilfan A, Duke T. Instabilities in the transient response of muscle. Biophys J. 2003;85(2):818–827.

[8] Stam S, Alberts J, Gardel ML, Munro E. Isoforms Confer Characteristic Force Generation and Mechanosensation by Myosin II Filaments. Biophys J. 2015;108(8):1997–2006.

[9] Kovács M, Wang F, Hu A, Zhang Y, Sellers JR. Functional divergence of human cytoplasmic myosin II. Kinetic characterization of the non-muscle IIA isoform. J Biol Chem. 2003;278(40):38132–38140.

[10] Guo B, Guilford WH. Mechanics of actomyosin bonds in different nucleotide states are tuned to muscle contraction. Proc Natl Acad Sci. 2006;103:9844–9849.

[11] Hill AV. The mechanical efficiency of frog's muscle. Proc R Soc London B. 1939;127(849):434–451.

[12] Ferrer JM, Lee H, Chen J, Pelz B, Nakamura F, Kamm RD, et al. Measuring molecular rupture forces between single actin filaments and actin-binding proteins. Proc Natl Acad Sci. 2008;105(34):9221–9226.

[13] Didonna Ba, Levine AJ. Unfolding cross-linkers as rheology regulators in F-actin networks. Phys Rev E. 2007;75(4):1–10.

[14] Wachsstock DH, Schwartz WH, Pollard TD. Affinity of alpha-actinin for actin determines the structure and mechanical properties of actin filament gels. Biophys J. 1993;65(July):205–214.

[15] Ott A, Magnasco M, Simon A, Libchaber A. Persistence of Actin. Macromolecules. 1993;48(3):1642.

[16] Kojima H, Ishijima A, Yanagida T. Direct measurement of stiffness of single actin filaments with and without tropomyosin by in vitro nanomanipulation. Proc Natl Acad Sci. 1994;91(26):12962–12966.

[17] Peskin CS, Odell GM, Oster GF. Cellular Motions and Thermal Fluctuations: The Brownian Ratchet. Biophys J. 1993;65(1):316–324.
