# Supplementary material for: MEDYAN: Mechanochemical Simulations of Contraction and Polarity Alignment in Actomyosin Networks
Source: PLoS Comput Biol. 2016 Apr 27;12(4):e1004877. doi: 10.1371/journal.pcbi.1004877 (PMC4847874; doi:10.1371/journal.pcbi.1004877)
Supplement: S4 Text — (A) Validation of the optimized NRM for stochastic reaction-diffusion, and benchmarking performance of the original Gillespie algorithm compared to the optimized NRM in typical cytoskeletal systems. (B) Validation of the cylindrical coarse-graining scheme, with benchmarking performance tests in cases of typical cytoskeletal systems. (C) A note on the computational time used for the various simulations shown in the Results section, and time comparisons to previous models. (PDF) [file pcbi.1004877.s004.pdf]

Supporting Information for

MEDYAN: Mechanochemical Simulations of Contraction and Polarity Alignment in Actomyosin Networks

Konstantin Popov, James Komianos, and Garegin Papoian

Benchmarking and validation of MEDYAN.

It is noted that all benchmarking simulations were performed on Intel Xeon Ivy Bridge E5-4640v2 processors running at 2.20 GHz, with 25.6GB DDR3 memory at 1333 MHz, as provided by the Deepthought2 supercomputing cluster at the University of Maryland.

A) Benchmarking and validation of the NRM algorithm in cytoskeletal stochastic reaction-diffusion.

We present a brief benchmarking and validation test of the optimized *Next Reaction Method* (NRM) algorithm [1] for stochastic reaction-diffusion that is used in MEDYAN, which provides huge optimizations for sparse chemical reaction networks (i.e. loosely coupled chemical reactions across the simulation domain). The original Gillespie algorithm, which is usually known as the Gillespie direct method (DM), which is used in previous works to describe the reaction-diffusion processes of lamellipodia [2, 3] as well as filopodia [4–7] has been validated thoroughly in the papers mentioned, and as MEDYAN is built based on this original algorithm, we will not validate the correctness of this approach here.

To validate the correctness, as well as benchmark performance increases, of the NRM algorithm compared to the original Gillespie DM, we ran a set of simulations for a smaller $1 \times 1 \times 1$ μm^3 actin network with the same parameters as described in the Results section of the paper with both reaction-diffusion algorithms for 10 s of simulation time. These benchmarking systems had identical configurations to the systems in the Results section (20 μM of diffusing actin and 50 filaments), but do not contain α-actinin or non-muscle myosin IIA mini-filaments. Unless otherwise noted besides the previously mentioned changes, all parameters used in these test cases are identical in setup to the Results section, including compartment size and reaction constants. 4 trajectories were run for each configuration.

To validate the NRM algorithm, we compare the (i) the mean filament length over time for 10 s of network evolution, and (ii) the critical concentration over time for 10 s of network evolution, averaged over the 4 trajectories, using the NRM and original Gillespie DM method, as shown in Figs 1 and 2. We see excellent agreement between these algorithms, as well as reasonable critical

concentration value $[A_c]$ reached for both algorithms, which can be solved analytically by using the polymerization and depolymerization rates of actin filaments in the simulation:

$$[A_c] = \frac{k_{\text{actin,depoly,+}} + k_{\text{actin,depoly,-}}}{k_{\text{actin,poly,+}} + k_{\text{actin,poly,-}}} = 0.17\mu M. \tag{1}$$

We anticipate our critical concentration value in simulation to be higher than this analytical value, as the mechanochemical effects included in this simulation (Brownian ratchet model of polymerizing filaments) disallow polymerizing filaments near a boundary to grow rapidly. We do, in fact, see a critical concentration reached in both algorithms of about 0.4 μM.

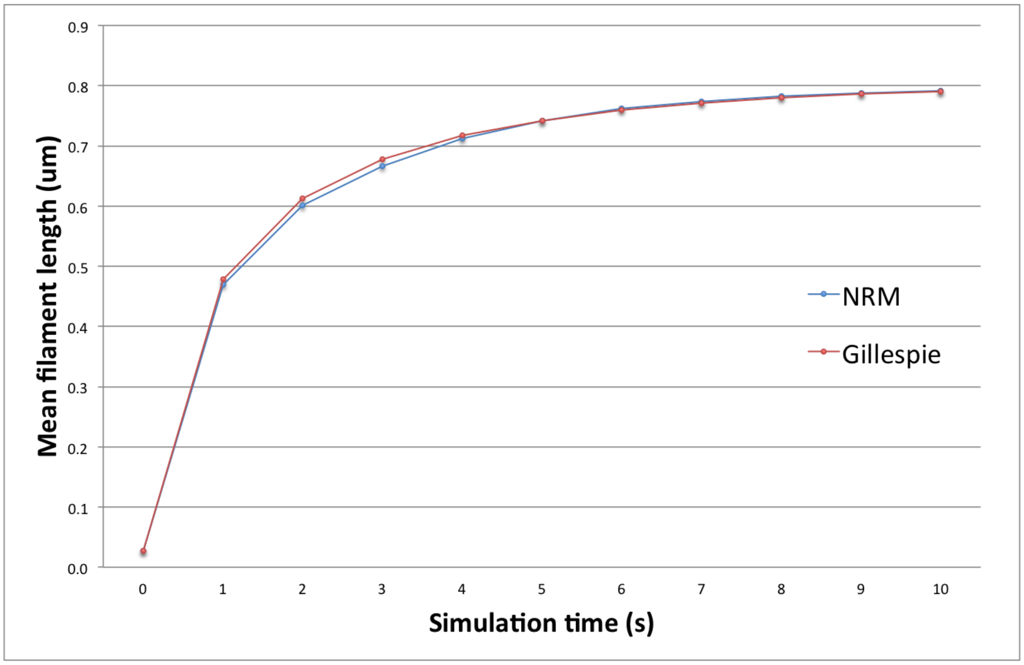

Figure 1: **Mean filament length over the 10 s of network evolution, using the NRM and Gillespie DM.** The algorithms are in excellent agreement.

To measure performance, we can look at the computation time for the chemical stochastic reaction-diffusion elapsed over the 10 s of simulation time, using both chemical algorithms, as shown in Fig 3. It is noted that this measurement ignores any mechanical equilibration steps, which would add additional time to the overall simulation. We see that the NRM algorithm outperforms the Gillespie DM by over 100-fold for this actin network. Larger $3 \times 3 \times 3$ μm^3 actin networks were not benchmarked, as the Gillespie DM does not achieve 1 s of simulation time within 12 hours of computation time.

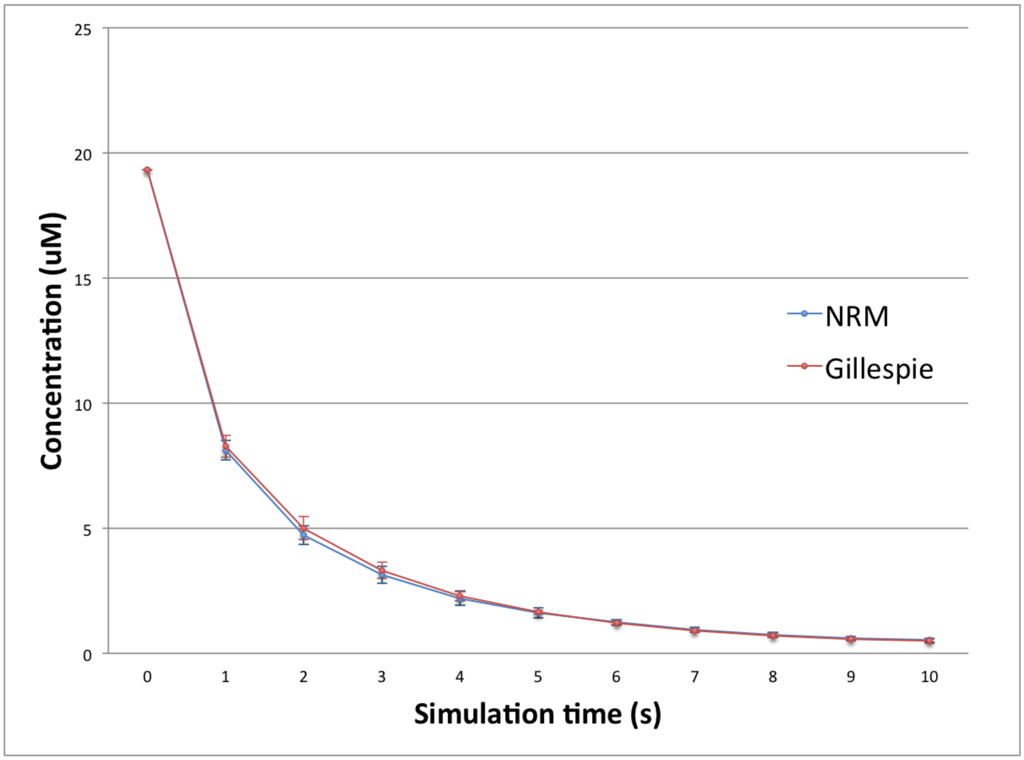

Figure 2: **Concentration of diffusing actin over the 10 s of network evolution for the actin system using the NRM and Gillespie DM.** The algorithms are in excellent agreement.

B) Benchmarking and validation of the coarse-grained polymer scheme in cytoskeletal systems.

We present a brief benchmarking and validation of the coarse-grained polymer scheme based on cylindrical monomer segments used in MEDYAN to simulate cytoskeletal filaments. The bead-spring model of semi-flexible polymers to represent individual monomeric components has been used by many computational works and has been proven as a correct description of polymer chain mechanics, so we will benchmark our coarse-grained cylindrical scheme by comparing directly to the bead-spring monomeric model with gradient tolerance $g_{\text{tol}} = 1\ pN$, which is, in our model, the limit of cylinder segment length approaching a single monomer size.

To validate and test performance of various levels of cylindrical coarse-graining compared to a simple bead-spring monomer model, we equilibrated a single filament with length $L = 1\ \mu m$ with fixed endpoints undergoing a point force of 50 pN at its center under varying equilibration parameter values and coarse-graining levels. Unless otherwise noted as changed, mechanical parameters for these filaments were taken as in the actin filament description in the Results section. It is, however, noted that the bending energy used between cylindrical segments scales inversely with the length of the cylindrical segments used, providing an accurate overall persistence length of the filament.

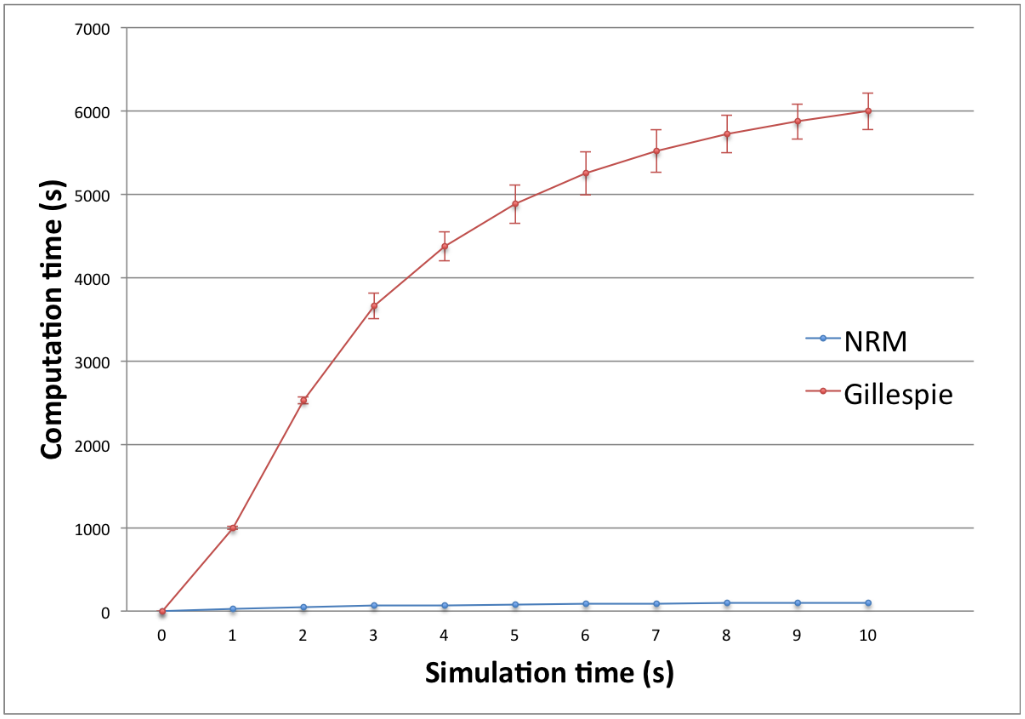

Figure 3: **Benchmarking of the NRM algorithm for stochastic reaction-diffusion.** There is a performance increase of over 100-fold for this actin system when using the NRM algorithm.

Figs 4 and 5 shows the various validation and benchmarking results. In Fig 4, the displacement error of the filament after mechanical equilibration e_d is shown for various coarse-grained cylindrical segment lengths l_{cyl} and gradient minimization tolerances g_{tol}, which we define as:

$$e_d = \frac{|x_{bs} - x_{cg}|}{L} \tag{2}$$

where x_{bs} represents the center point of the filament ($L/2$ along the length of the filament) after equilibration in the bead-spring model, and x_{cg} is the same center point after equilibration with a coarse-grained cylindrical description.

We then performed a similar validation and benchmarking for a microtubule-like filament as also shown in Figs 4 and 5, which has an estimated persistence length of 5 mm and an estimated monomer size of 2 nm. These parameters defined the bending energy used in the harmonic bending potential for the microtuble, which is increased in comparison to the bending energy of an actin filament cylindrical segment by a factor of over 1000. This benchmarking shows that a polymer with high stiffnesses can be simulated efficiently and accurately with this approach. As in the actin filament example, the bending energy used scales inversely with the length of the chosen cylindrical segment.

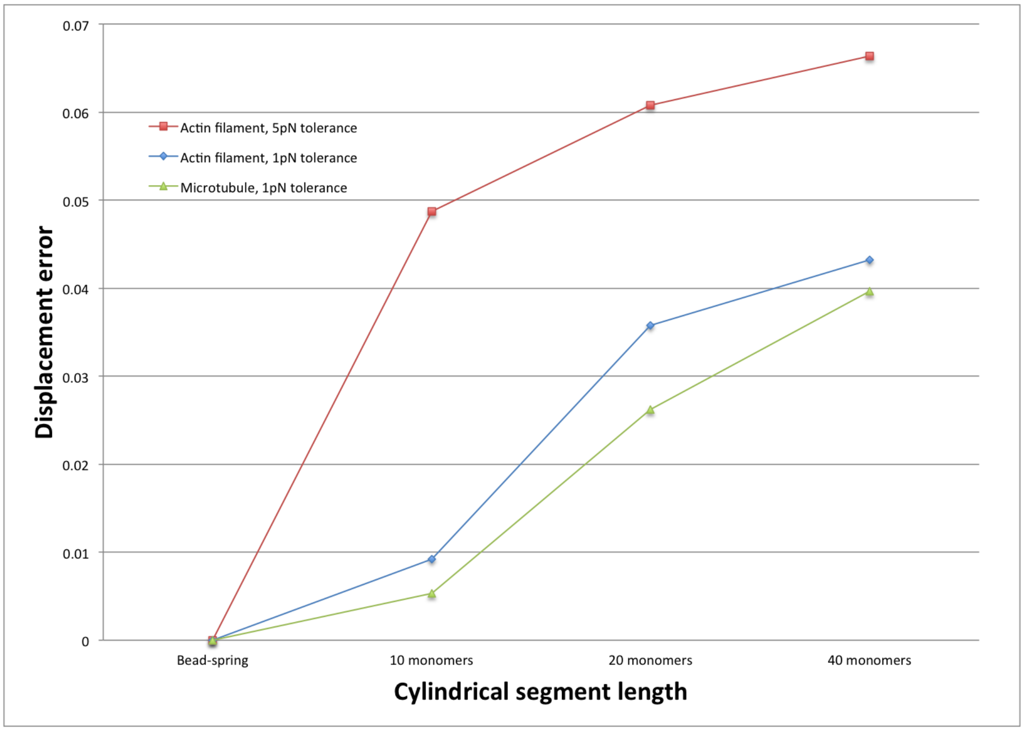

Figure 4: **Polymer displacement error under various coarse-graining parameters and tolerance levels.** Since this error compares to a bead-spring description, there is no error shown for the bead-spring model. Displacement errors stay under 5% of the total polymer length for all levels of coarse-graining used.

C) A note on computational performance for simulations in Results section, and time comparisons to previous models.

Computation time used for simulations in the Results section was varying depending on the size and concentrations used in the various actomyosin networks. For a moderate-concentration actomyosin system in 1 μm^3 of simulation volume, 2000 s of simulation time was reached in about 12 hours. For increasing concentrations in this smaller domain, computation time was increased up to 2 days. The larger actomyosin systems, which contain 27 μm^3 of simulation volume, ran to 500 s of simulation time in about 6 days. It is noted that these performances measured are serial computations, as a parallel MEDYAN implementation has not yet been created.

In estimating performance increases of MEDYAN from previous versions of the model, the code of [2] was typically benchmarked to run 1 s of simulation with reasonable concentrations of actin in a few micron sized domain in about 6 hours of computation time.

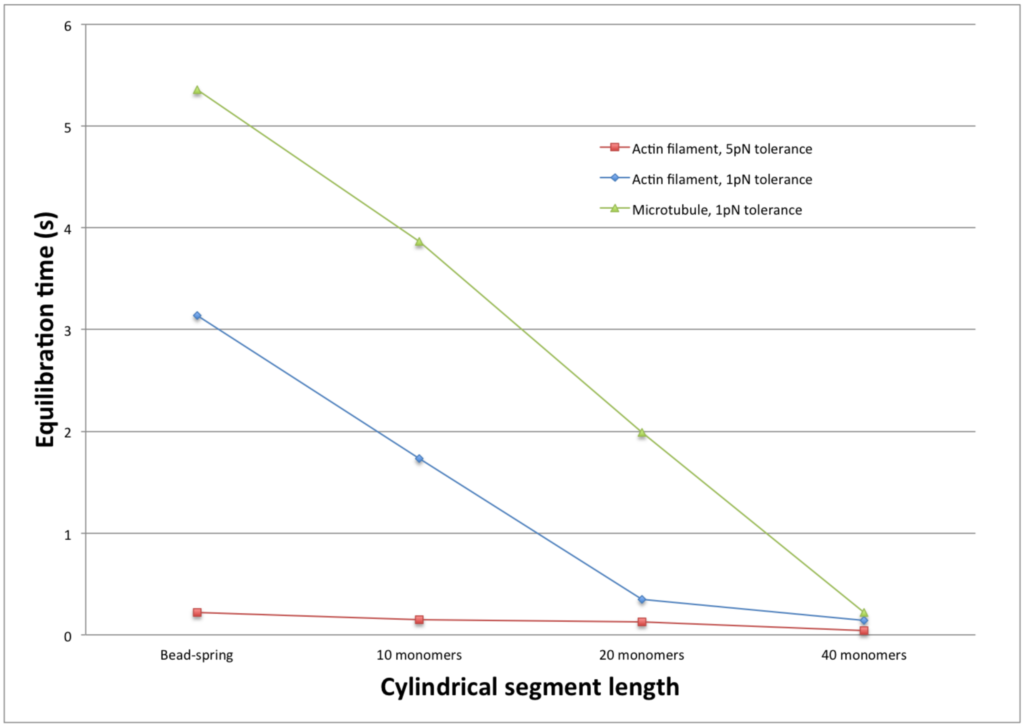

Figure 5: **Benchmarking polymer equilibration time under various coarse-graining parameters and gradient tolerance levels.** Equilibration speed ups are more apparent for the lower tolerance levels. Microtubules show similar equilibration times compared to actin filaments, and could probably benefit from even more coarse-graining.

References

[1] Gibson MA, Bruck J. Efficient Exact Stochastic Simulation of Chemical Systems with Many Species and Many Channels. J Phys Chem A. 2000;104:1876–1889.

[2] Hu L, Papoian GA. Molecular transport modulates the adaptive response of branched actin networks to an external force. J Phys Chem B. 2013;117(42):13388–13396.

[3] Hu L, Papoian GA. Mechano-chemical feedbacks regulate actin mesh growth in lamellipodial protrusions. Biophys J. 2010;98(8):1375–1384.

[4] Zhuravlev PI, Lan Y, Minakova MS, Papoian GA. Theory of active transport in filopodia and stereocilia. Proc Natl Acad Sci. 2012;109(27):10849–10854.

[5] Zhuravlev PI, Papoian GA. Protein fluxes along the filopodium as a framework for understanding the growth-retraction dynamics: the interplay between diffusion and active transport. Cell Adh Migr. 2011;5(5):448–456.

[6] Zhuravlev PI, Papoian GA. Molecular noise of capping protein binding induces macroscopic instability in filopodial dynamics. Proc Natl Acad Sci. 2009;106(28):11570–11575.

[7] Lan Y, Papoian GA. The stochastic dynamics of filopodial growth. Biophys J. 2008;94(10):3839–3852.
