# Supplementary material for: MEDYAN: Mechanochemical Simulations of Contraction and Polarity Alignment in Actomyosin Networks
Source: PLoS Comput Biol. 2016 Apr 27;12(4):e1004877. doi: 10.1371/journal.pcbi.1004877 (PMC4847874; doi:10.1371/journal.pcbi.1004877)
Supplement: S1 Table — These models are described in terms of their mechanochemical representations of cytoskeletal dynamics as outlined in the Introduction section. (PDF) [file pcbi.1004877.s005.pdf]

Supporting Information for

MEDYAN: Mechanochemical Simulations of Contraction and Polarity Alignment in Actomyosin Networks

Konstantin Popov, James Komianos, and Garegin Papoian

A comparison of MEDYAN to other agent-based cytoskeletal models.

This table is shown on the next page, with comparison categories as discussed in the Introduction section. Those categories are organized as follows:

- **Chemistry** - The implementation of cytoskeletal chemistries within the model, which could include (but not limited to) filament, cross-linker, and molecular motor chemical dynamics.
- **Mechanics** - The force-field description of semi-flexible filaments in the model, as well as other mechanical elements, including cross-linkers and molecular motors.
- **Diffusion and active transport** - The capability of the model to include diffusion and active transport of some or all of its cytoskeletal species.
- **Mechanochemical feedback** - The model's capability to include force-dependent reaction rates for some or all of its cytoskeletal species, which are updated dynamically during runtime.
- **Dimensionality, time and length scales** - The previous time and length scales achieved by the model in its publications.
- **Public code availability** - The access of source code and/or example applications for the model in a public repository.

Model	Chemistry	Mechanics	Diffusion and active transport	Mechanochemical feedback	Dimensionality, time and length scales	Public code availability	Refs
SCPR, Bidone et al. 2014, Tang et al. 2014, Laporte et al. 2012, Wang et al. 2008	• Stochastic growth of actin filaments<br>• Binding of cross-linkers and motors from bulk reservoir<br>• No filament branching reported	• Langevin dynamics with a coarse-grained point-like representation for filaments<br>• Actin filament bending and stretching, motor and cross-linker interaction potentials<br>• No inter-filament excluded volume effects reported	• Filaments diffuse via Brownian motion	None reported	• 2D, 3D<br>• 1000 s timescales<br>• 5-15 μm length scales	None reported	[1–4]
Cytosim, Nédélec and Foethke 2002-2007	• Stochastic growth of filaments<br>• Binding of cross-linkers and motors from bulk reservoir<br>• Filament branching	• Langevin dynamics with a coarse-grained point-like representation for filaments<br>• Interaction potentials for filaments, cross-linkers, motors, branching molecules, in various verisons.<br>• Repulsion or attraction between filament points for excluded volume effects	• Filaments diffuse via Brownian motion	• Mechanochemical models for filaments, cross-linkers, motors	• 2D, 3D<br>• Time and length scales vary greatly depending on simulated system	• Example applications available for download at www.cytosim.org	[5, 6]
Jung et al 2015, Kim et al. 2009-2014	• Stochastic growth of actin filaments<br>• Binding of cross-linkers from bulk reservoir<br>• Stochastic myosin II filament binding and walking	• Langevin dynamics with a coarse-grained cylindrical segmentation for actin filaments<br>• Cross-linker interaction potentials and very detailed myosin II potentials<br>• Nearest-distance repulsion for cylinder excluded volume	• Filaments diffuse via Brownian motion<br>• Myosin II filaments diffuse via Brownian motion<br>• Actin monomers and cross-linkers do not diffuse	• Detailed mechanochemical models for cross-linker unbinding, and myosin II filament walking and unbinding	• 3D<br>• 200 s timescales<br>• 2-5 μm length scales	None reported	[7–9]
Muller et al. 2015, Cyron et al. 2009-2013	• Cross-linker spatially resolved binding onto filaments<br>• No growth or nucleation of filaments<br>• Does not implement filament branching	• Finite-element Brownian dynamics of semi-flexible filament bending and stretching<br>• Cross-linker interaction potentials<br>• Inter-filament excluded volume not reported in detail	• Filaments and cross-linkers diffuse via Brownian motion<br>• Actin monomers do not diffuse	None reported	• 3D<br>• 1000 s timescales<br>• 1-10 μm length scales	None reported	[10–13]
Odell et al. 2008	• Spatially resolved, stochastic chemical reactions via cytoplasmic domains<br>• Mass-action kinetic equations for chemical reactions within cytoplasmic domains<br>• Stochastic nucleation and growth of microtubules	• Coarse-grained microtubules by spring segments, with bending and stretching potentials<br>• No inter-filament excluded volume reported	• Continuum diffusive flux between cytoplasmic domains<br>• Convective transport modeled by viscous drag force on cytoplasmic domains	• Mechanochemical model for microtubules	• 3D<br>• 500 s timescales<br>• 80 μm length scales	• Example application available for download at www.celldynamics.org	[14]
Rafelski et al. 2008, Alberts et al. 2004	• Spatially resolved, stochastic chemical reactions via partial differential equation reaction-diffusion scheme<br>• Stochastic branching, nucleation and growth of actin filaments<br>• Stochastically varying biochemical states of filament	• Brownian dynamics of branched, rigid actin filaments and collision interactions with surfaces<br>• No inter-filament excluded volume reported	• Partial differential equation reaction-diffusion scheme for actin monomers and branching molecules	None reported	• 3D<br>• 100 s timescales<br>• 10 μm length scales	• Source code and example application available for download at www.celldynamics.org	[15, 16]
MEDYAN	• Stochastic reaction-diffusion master equation<br>• Stochastic, spatially resolved filament nucleation and branching, growth, and state transitions<br>• Cross-linker and motor dynamics	• Conjugate gradient energy minimization<br>• Interaction potentials for cross-linkers, motors, branching molecules, and filaments<br>• Novel cylindrical rigid body repulsion	• Stochastic, compartment based reaction-diffusion scheme for general transport of any unbound cytosolic species<br>• No Brownian motion of filaments	• Detailed mechanochemical models for filaments, cross-linkers, and motors	• 3D<br>• 2000 s timescales<br>• 1-3 μm length scales	• Source code and example applications available for download at www.medyan.org	-

References

[1] Bidone TC, Tang H, Vavylonis D. Dynamic Network Morphology and Tension Buildup in a 3D Model of Cytokinetic Ring Assembly. Biophys J. 2014;107(11):2618–2628.

[2] Tang H, Laporte D, Vavylonis D. Actin cable distribution and dynamics arising from cross-linking, motor pulling and filament turnover. Mol Biol Cell. 2014;25(19):3006–3016.

[3] Laporte D, Ojkic N, Vavylonis D, Wu JQ. Alpha-Actinin and fimbrin cooperate with myosin II to organize actomyosin bundles during contractile-ring assembly. Mol Biol Cell. 2012;23(16):3094–3110.

[4] Wang H, Vavylonis D. Model of For3p-Mediated Actin Cable Assembly in Fission Yeast. PLoS One. 2008;3(12):e4078.

[5] Nédélec F. Computer simulations reveal motor properties generating stable antiparallel microtubule interactions. J Cell Biol. 2002;158(6):1005–1015.

[6] Nédélec F, Foethke D. Collective Langevin dynamics of flexible cytoskeletal fibers. New J Phys. 2007;9(11):427.

[7] Kim T. Determinants of contractile forces generated in disorganized actomyosin bundles. Biomech Model Mechanobiol. 2014;14(2):345–355.

[8] Kim T, Gardel ML, Munro ED. Determinants of Fluidlike Behavior and Effective Viscosity in Cross-Linked Actin Networks. Biophys J. 2014;106(3):526–534.

[9] Jung W, Murrell MP, Kim T. F-actin cross-linking enhances the stability of force generation in disordered actomyosin networks. Comput Part Mech. 2015;2(4):317–327.

[10] Müller KW, Cyron CJ, Wall WA. Computational analysis of morphologies and phase transitions of cross-linked, semi-flexible polymer networks. Proc R Soc London A. 2015;471(2182):20150332.

[11] Cyron CJ, Müller KW, Schmoller KM, Bausch AR, Wall WA, Bruinsma RF. Equilibrium phase diagram of semi-flexible polymer networks with linkers. Europhys Lett. 2013;102(3):38003.

[12] Cyron CJ, Müller KW, Bausch AR, Wall Wa. Micromechanical simulations of biopolymer networks with finite elements. J Comput Phys. 2013;244:236–251.

[13] Cyron CJ, Wall WA. Finite-element approach to Brownian dynamics of polymers. Phys Rev E. 2009;80(6):1–12.

[14] Odell GM, Foe VE. An agent-based model contrasts opposite effects of dynamic and stable microtubules on cleavage furrow positioning. J Cell Biol. 2008;183(3):471–483.

[15] Rafelski SM, Alberts JB, Odell GM. An experimental and computational study of the effect of ActA polarity on the speed of Listeria monocytogenes actin-based motility. PLoS Comput Biol. 2009;5(7):e1000434.

[16] Alberts JB, Odell GM. In silico reconstitution of Listeria propulsion exhibits nano-saltation. PLoS Biol. 2004;2(12):e412.
