# Supplementary material for: MEDYAN: Mechanochemical Simulations of Contraction and Polarity Alignment in Actomyosin Networks
Source: PLoS Comput Biol. 2016 Apr 27;12(4):e1004877. doi: 10.1371/journal.pcbi.1004877 (PMC4847874; doi:10.1371/journal.pcbi.1004877)
Supplement: S2 Table — (A) Reaction rates, taken from experimental kinetic data. (B) Mechanical parameters, chosen to mimic actomyosin mechanics. (C) Mechanochemical parameters, adopted from single molecule experiments and the Parallel Cluster Model. (D) Other general parameters and their description. (PDF) [file pcbi.1004877.s006.pdf]

Supporting Information for
MEDYAN: Mechanochemical Simulations of Contraction and Polarity Alignment in Actomyosin Networks

Konstantin Popov, James Komianos, and Garegin Papoian

Simulation parameters used in the actomyosin systems.

Table 2A: Reaction rates.

**Reaction**	**Symbol**	**Value [for simulation]***	**Reference**
Actin diffusion	$k_{\text{actin, diff}}$	$20\ \mu m^2 \cdot s^{-1}$ [$80\ s^{-1}$] **a**	-
α-actinin diffusion	$k_{\alpha,\text{diff}}$	$k_{\text{actin, diff}}/10\ s^{-1}$ **a**	-
NMIIA mini-filament diffusion	$k_{\text{NMIIA, diff}}$	$k_{\text{actin, diff}}/100\ s^{-1}$ **a**	-
Actin polymerization at plus end	$k_{\text{actin,poly},+}$	$11.6\ \mu M^{-1}s^{-1}$ [$0.151\ s^{-1}$] **b**	[1]
Actin polymerization at minus end	$k_{\text{actin,poly},-}$	$1.3\ \mu M^{-1}s^{-1}$ [$0.017\ s^{-1}$] **b**	[1]
Actin depolymerization at plus end	$k_{\text{actin,depoly},+}$	$1.4\ s^{-1}$	[1]
Actin depolymerization at minus end	$k_{\text{actin,depoly},-}$	$0.8\ s^{-1}$	[1]
NMIIA head binding	$k_{\text{NMIIA,bind}}$	$0.2\ s^{-1}$	[2]
NMIIA head unbinding under no external load	$k^0_{\text{NMIIA,unbind}}$	$1.7\ s^{-1}$ **c**	-
α-actinin binding	$k_{\alpha,\text{bind}}$	$0.7\ \mu M^{-1}s^{-1}$ [$0.009\ s^{-1}$]	[3]
α-actinin unbinding under no external load	$k^0_{\alpha,\text{unbind}}$	$0.3\ s^{-1}$	[3]

*****- The value in brackets is the constant used in simulation, calculated based on a compartment volume of $(500\ nm)^3$.

a- This calculation can be seen in Section A of S2 Text.

b- All polymerization rates specified are assumed to be under no external load.

c- This rate was calculated based on $k_{\text{NMIIA,bind}}$, determined by Kovacs et al. [2], as well as the known duty ratio of NMIIA that was also confirmed in the same work.

Table 2B: Mechanical parameters.

Parameter	Symbol	Value	Reference
Length of cylindrical actin filament segment	l_{cyl}	27 nm, 108 nm [a]	[4]
Actin filament bending energy	$\varepsilon_{\text{actin,bend}}$	2690 $pN \cdot nm$, 672 $pN \cdot nm$ [b]	[4]
Actin filament stretching constant	$K_{\text{actin,str}}$	100 pN/nm [c]	-
Actin filament excluded volume repulsion constant	$K_{\text{actin,vol}}$	10^5 pN/nm^4 [d]	-
NMIIA head stretching constant	$K_{\text{NMIIA,stretching}}$	2.5 pN/nm [e]	[5]
α-actinin stretching constant	$K_{\alpha,\text{stretching}}$	8 pN/nm	[6]
Boundary repulsion energy	$\varepsilon_{\text{boundary}}$	$k_{\text{b}}T$, 4.1 $pN \cdot nm$	[7]
Boundary repulsion screening length	$\lambda_{\text{boundary}}$	2.7 nm	[7]

[a]- The two values shown are for the different l_{cyl} lengths used in the smaller and larger system simulations; a more aggressive coarse-graining scheme was used in the latter.

[b]- Calculated based on the persistence length of actin filaments, which was determined by Ott et al. [4]. The bending energy for a cylinder with l_{cyl} (= 27 or 108 nm) in our system was calculated as $\varepsilon_{\text{actin,bending}} = l_{\text{p}} \cdot k_{\text{b}}T/l_{\text{cyl}}$ where l_{p} is the persistence length of an actin filament. The two values shown are for the l_{cyl} lengths used in the smaller and larger system simulations.

[c]- This value was chosen to be smaller than the known stretching rigidity of actin filaments, experimentally determined by Kojima et al. [8]. This allowed for higher computational efficiency in this study. [9, 10] also make similar assumptions, and have shown that this affects actomyosin dynamics very little in both models. While this constant is low, in comparison to other mechanical constants in the system it is still sufficiently high such that cylinder linear deformations are extremely small in simulation (the fraction of cylinders that are linearly stretched or compressed beyond 10% of their equilibrium length after mechanical minimization is zero in all simulated cases).

[d]- Chosen to mimic rigid body repulsion, keeping in mind that the diameter of an actin filament is about 6 nm [11].

[e] -This value will be multiplied by the number of heads in the NMIIA ensemble to obtain a minifilament stretching constant.

Table 2C: Mechanochemical parameters.

Parameter	Symbol	Value	Reference
Duty ratio of NMIIA head	ρ	0.1	[2]
Unbinding force of NMIIA head	$F_{\mathrm{NMIIA,unbind}}$	12.6 pN	[12]
Stall force of NMIIA head	F_{stall}	15 pN [a]	-
NMIIA binding kinetics parameter	α	1.0 [b]	-
NMIIA unbinding kinetics parameter	β	0.2 [b]	-
NMIIA bound heads force dependence parameter	γ	$0.05/pN$ [b]	-
NMIIA stall force dependence parameter	ζ	0.1 [b]	-
Characteristic unbinding force of α-actinin	$F_{\alpha,\mathrm{unbind}}$	17.2 pN	[13]
Characteristic polymerization force of actin filaments	$F_{\mathrm{actin,poly}}$	1.5 pN	[14]

[a]- Calculated as $K_{\mathrm{NMIIA,stretching}} \cdot d_{\mathrm{NMIIA,step}}$.

[b]- Chosen to produce realistic attachment and walking times for NMIIA ensembles under zero load, as well as stall loads. See section A of S3 Text for more details on this parameter fitting.

Table 2D: Other general parameters.

Parameter	Symbol	Value
Number of compartments in each dimension	N	2
Compartment length	l_{comp}	500 nm [a]
Gradient minimization tolerance	g_{tol}	1 pN [b]
Timestep between mechanical equilibrations	$t_{\mathrm{minimization}}$	0.1 s [c]

[a]- Choosing this compartment length is dependent on the Kuramoto length of the actomyosin systems, which is derived in Section A of S2 Text.

[b]- Choosing the gradient minimization tolerance is discussed in Section B of S2 Text.

[c]- Choosing the timestep between mechanical equilibrations is discussed in Section C of S2 Text.

References

[1] Fujiwara I, Vavylonis D, Pollard TD. Polymerization kinetics of ADP- and ADP-Pi-actin determined by fluorescence microscopy. Proc Natl Acad Sci. 2007;104(21):8827–8832.

[2] Kovács M, Wang F, Hu A, Zhang Y, Sellers JR. Functional divergence of human cytoplasmic myosin II. Kinetic characterization of the non-muscle IIA isoform. J Biol Chem. 2003;278(40):38132–38140.

[3] Wachsstock DH, Schwartz WH, Pollard TD. Affinity of alpha-actinin for actin determines the structure and mechanical properties of actin filament gels. Biophys J. 1993;65(July):205–214.

[4] Ott A, Magnasco M, Simon A, Libchaber A. Persistence of Actin. Macromolecules. 1993;48(3):1642.

[5] Vilfan A, Duke T. Instabilities in the transient response of muscle. Biophys J. 2003;85(2):818–827.

[6] Didonna Ba, Levine AJ. Unfolding cross-linkers as rheology regulators in F-actin networks. Phys Rev E. 2007;75(4):1–10.

[7] Hu L, Papoian GA. Molecular transport modulates the adaptive response of branched actin networks to an external force. J Phys Chem B. 2013;117(42):13388–13396.

[8] Kojima H, Ishijima A, Yanagida T. Direct measurement of stiffness of single actin filaments with and without tropomyosin by in vitro nanomanipulation. Proc Natl Acad Sci. 1994;91(26):12962–12966.

[9] Kim T. Determinants of contractile forces generated in disorganized actomyosin bundles. Biomech Model Mechanobiol. 2014;14(2):345–355.

[10] Tang H, Laporte D, Vavylonis D. Actin cable distribution and dynamics arising from cross-linking, motor pulling and filament turnover. Mol Biol Cell. 2014;25(19):3006–3016.

[11] Splettstoesser T, Holmes KC, Noé F, Smith JC. Structural modeling and molecular dynamics simulation of the actin filament. Proteins Struct Funct Bioinforma. 2011;79(7):2033–2043.

[12] Erdmann T, Albert PJ, Schwarz US. Stochastic dynamics of small ensembles of non-processive molecular motors: The parallel cluster model. J Chem Phys. 2013;139(17):175104.

[13] Ferrer JM, Lee H, Chen J, Pelz B, Nakamura F, Kamm RD, et al. Measuring molecular rupture forces between single actin filaments and actin-binding proteins. Proc Natl Acad Sci. 2008;105(34):9221–9226.

[14] Footer M, Kerssemakers J, Theriot J, Dogterom M. Direct measurement of force generation by actin filament polymerization using an optical trap. Proc Natl Acad Sci. 2007;104(7):2181–2186.
