# Supplementary figures and images for: MEDYAN: Mechanochemical Simulations of Contraction and Polarity Alignment in Actomyosin Networks
Source: PLoS Comput Biol. 2016 Apr 27;12(4):e1004877. doi: 10.1371/journal.pcbi.1004877 (PMC4847874; doi:10.1371/journal.pcbi.1004877)

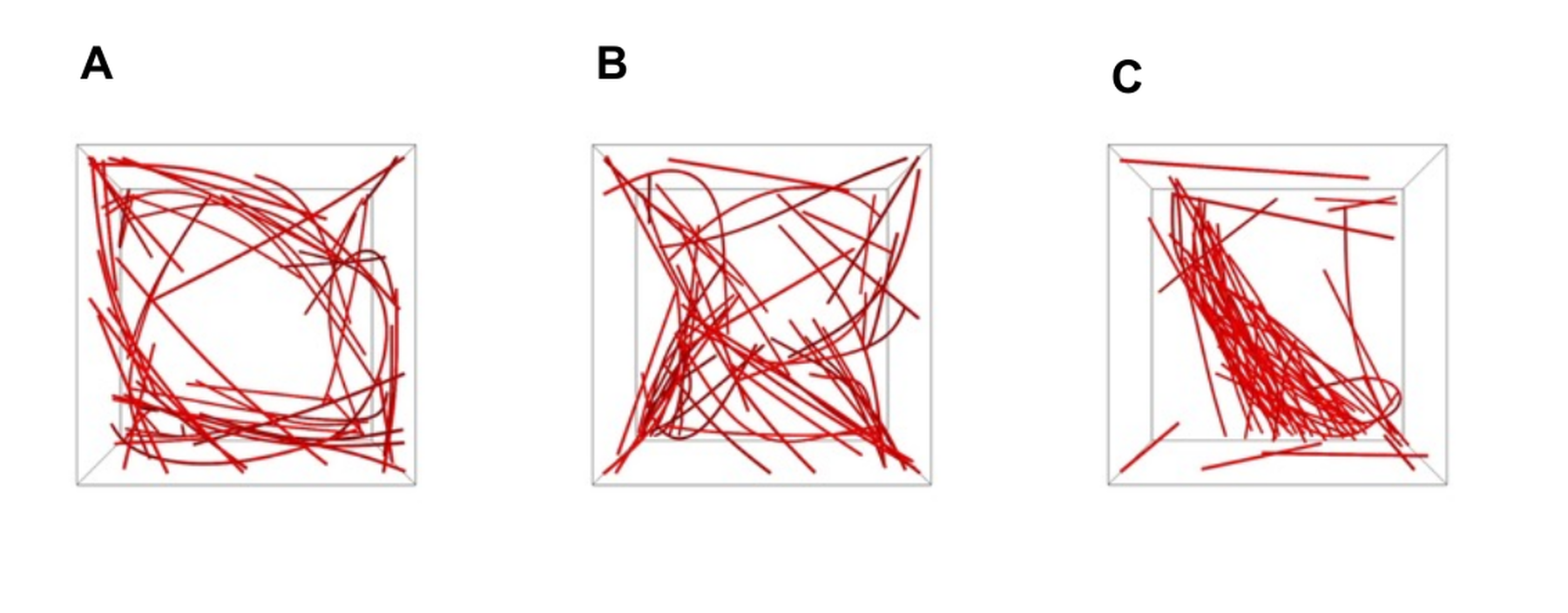

Supplement: S1 Fig — (A) A R g of 1.77 μm. (B) A R g of 1.51 μm. (C) A R g of 1.22 μm. Corresponding to a decrease of R g from left to right, the actomyosin networks show more contractile structure formation. These snapshots are shown without monomeric actin, α-actinin, and NMIIA mini-filaments for simplicity. (PNG) [file pcbi.1004877.s011.png]

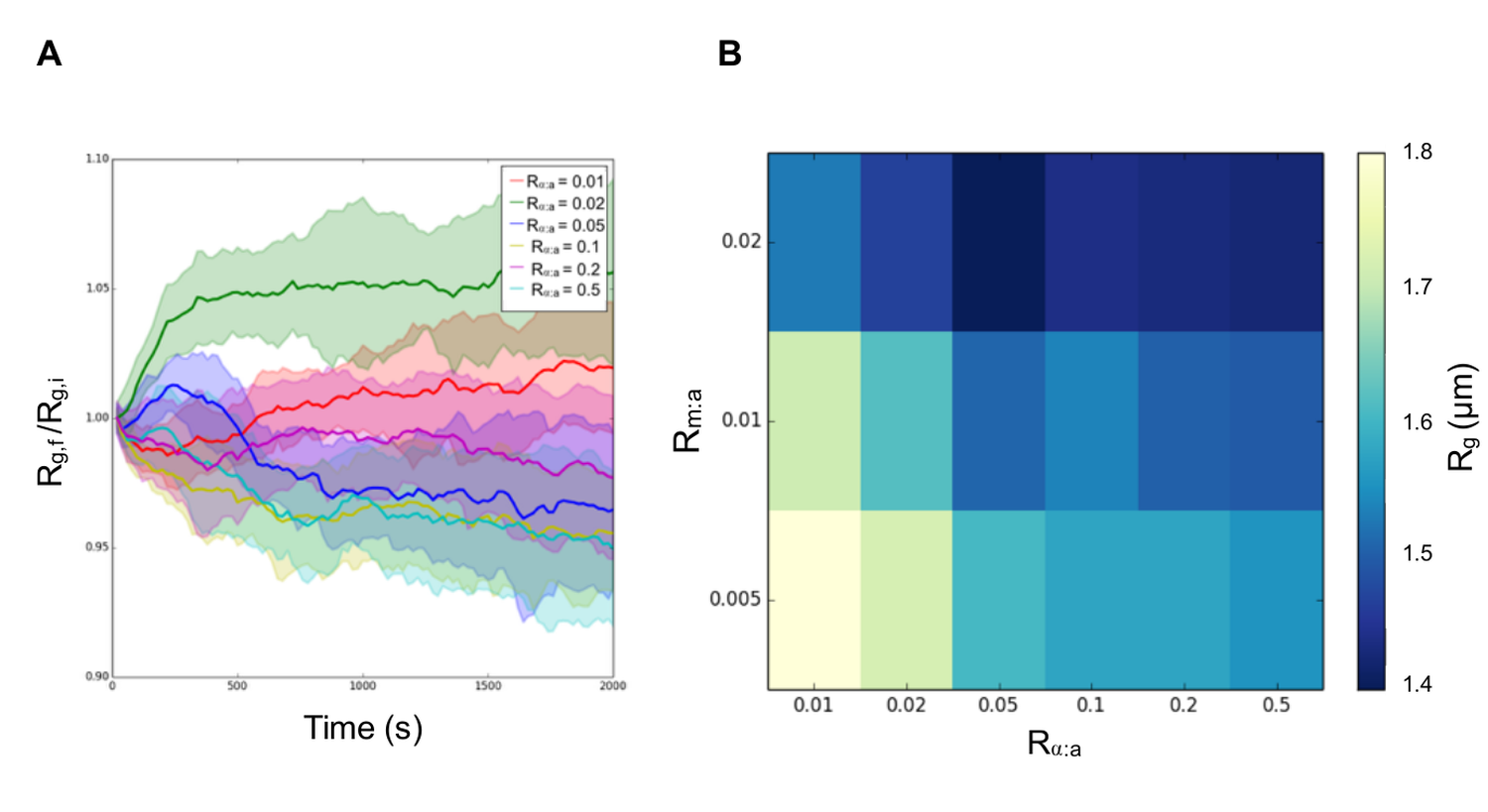

Supplement: S2 Fig — (A) A heat map of actomyosin network R g as a function of R m:a and R α:a after 2000 s of network evolution. (B) Actomyosin network R g,f/R g,i over time for various R α:a with fixed R m:a = 0.01. We see that in changing this modeling assumption, overall R g is reduced and its dependence on R α:a is altered. (PNG) [file pcbi.1004877.s012.png]
